# Association Between Prospective Registration and Quality of Systematic Reviews in Type 2 Diabetes Mellitus: A Meta-epidemiological Study

**DOI:** 10.3389/fmed.2021.639652

**Published:** 2021-06-28

**Authors:** Qiuyi Zheng, Fenghua Lai, Bin Li, Jia Xu, Jianyan Long, Sui Peng, Yanbing Li, Yihao Liu, Haipeng Xiao

**Affiliations:** ^1^Department of Endocrinology, The First Affiliated Hospital of Sun Yat-sen University, Guangzhou, China; ^2^Clinical Trials Unit, The First Affiliated Hospital of Sun Yat-sen University, Guangzhou, China

**Keywords:** meta-epidemiological study, registration, methodological quality, reporting quality, systematic reviews, type 2 diabetes mellitus

## Abstract

**Background:** We sought to investigate the methodological and reporting quality of published systematic reviews describing randomized controlled trials in type 2 diabetes mellitus and analyze their association with status of protocol registration.

**Methods:** We searched the PubMed database and identified non-Cochrane systematic reviews, with or without meta-analysis, reporting on type 2 diabetes mellitus and published between 2005 and 2018. We then randomly selected 20% of these reviews in each year, and performed methodological and reporting quality assessment using the Assessment of Multiple Systematic Review 2 (AMSTAR-2) checklist and Preferred Reporting Items for Systematic Reviews and Meta-analyses (PRISMA) statement. We also conducted regression analyses to explore the association between characteristics of systematic reviews and AMSTAR-2 or PRISMA scores.

**Results:** A total of 238 systematic reviews, including 33 registered and 205 non-registered articles, met the inclusion criteria and were subsequently reviewed. Analysis indicated an increase in both registered rates and quality of systematic reviews in type 2 diabetes mellitus over the recent years. With regards to methodological and reporting quality, we found higher scores in registered, relative to non-registered reviews (AMSTAR-2 mean score: 18.0 vs. 14.5, *P* = 0.000; PRISMA mean score: 20.4 vs. 17.6, *P* = 0.000). AMSTAR-2 and PRISMA scores were associated with registration status, country of the first author, and statistical results, whereas the proportion of discussing publication bias and reporting funding sources were <40% for both registered and non-registered systematic reviews.

**Conclusions:** Methodological and reporting quality of systematic reviews in type 2 diabetes mellitus indicates an improvement in the recent years. However, the overall quality remains low, necessitating further improvement. Future studies are expected to pay more attention to prospective registration, description of publication bias and reporting of funding sources.

## Introduction

Type 2 diabetes mellitus (T2DM) is one of the most common chronic diseases in the world (1). To explore safety and efficacy of new interventions for managing the disease, many randomized controlled trials (RCTs) have been conducted. However, many trials investigating the same intervention have reported conflicting results, necessitating systematic reviews (SRs) and meta-analyses.

The publication of articles reporting SRs were about 2,500 in 2004 (2), and by 2014 they had increased by 3-fold to more than 8,000 (3). However, methodological and reporting quality in many of these reviews and meta-analyses remain unclear. Key concerns include a lack of reporting complete of methods and contacting authors for unpublished data, as well as use of inappropriate statistical methods (3).

To promote transparency and coordination of non-Cochrane SRs, an International Prospective Register of Systematic Reviews (PROSPERO) was established in 2011. This free online facility offers registration and public access to non-Cochrane SRs (4), with reports indicating that prospective registration therein may improve the quality of SRs. Resulting SR protocols help to define the study purpose, inclusion criteria, methods, data analysis, thereby avoid reporting bias during the research process (5, 6).

To date, no specific study has reported on the quality of SRs in T2DM. Therefore, we aimed to investigate the methodological and reporting quality of SRs describing RCTs in T2DM over the last few years, and analyze its association with status of protocol registration. Furthermore, we explored potential aspects for improving SRs quality on RCT in T2DM, which may provide some advice for future reviewers.

## Methods

### Search Strategy

In order to gain a snapshot of the literature and explore potential differences before/after the introduction of PROSPERO, we restricted the search to PubMed and the years 2005-2018. On 24th September, 2019, we searched the PubMed database, using the following strategy: (((((RCTs [Title/Abstract] OR randomized [Title/Abstract] OR randomized [Title/Abstract])) AND (systematic [Title/Abstract] OR meta [Title/Abstract] OR meta-analysis [Title/Abstract])) AND (type 2 diabetes [Title/Abstract] OR type II diabetes [Title/Abstract] OR T2DM [Title/Abstract]))) AND (“2005/01/01” [Date - Publication]: “2018/12/31” [Date - Publication]).

### Eligibility Criteria

SR articles, with or without meta-analysis data that met the following criteria were included in our study: (1) had RCTs that explored safety and efficacy of interventions related to T2DM; and (2) were published in English, between 2005 and 2018.

Conversely, those that met the following criteria were excluded: (1) diagnostic test accuracy review, meta-epidemiological study, update review or published as a thesis; (2) included type 1 diabetes mellitus, gestational diabetes mellitus or special types of diabetes mellitus; (3) were duplicates or had no full texts. We also excluded studies from Cochrane library. Despite protocol registration being necessary for Cochrane SRs, studies have reported that the quality of Cochrane-derived SRs is better than that of non-Cochrane SRs (3, 7–9), which may affect the results of our study.

### Screening and Selection

Two reviewers (QZ and FL) independently reviewed the titles and abstracts, before identifying and selecting potential eligible SRs. Currently, there are no recognized method for random selection of articles, but also no recognized sample size to detect the difference of methodological quality and reporting quality between registered and non-registered systematic reviews. In previous studies, certain number of studies were randomly selected from all eligible subjects, such as 50 (7) and 100 (8). However, the sampling method of these studies can't present good representation in each year. Therefore, based on the available time and other resources, we randomly selected the first 20% of studies in each year, by generating the random number tables in Microsoft Excel (Microsoft Corp, Redmond, WA, www.microsoft.com). If a selected SR was not eligible, following reading of the full text and according to the inclusion and exclusion criteria, a successive record was used to replace it (7). Any disagreements were resolved by a third reviewer (Y Liu).

### Data Extraction

Two reviewers (QZ and FL) independently extracted the following data from the eligible SRs: title, publication year, journal name, impact factor (IF) at the time of this study, country of the first author, with meta-analysis or not, registration status, intervention type, number of RCTs, number of included patients, statistical result (positive or negative). Any conflicts between them resolved by consensus.

### Assessment of Methodological and Reporting Quality

We evaluated the methodological quality of the included SRs using A Measurement Tool to Assess Systematic Reviews 2 (AMSTAR-2) tool (10), a widely cited tool (10, 11). The tool comprises 16 items, with 7 critical items (Items 2, 4, 7, 9, 11, 13, and 15) and 9 non-critical items (Items 1, 3, 5, 6, 8, 10, 12, 14, and 16) (Supplementary Table 1). Each item was judged as: “Yes” (item fully addressed), “Partial Yes” (item not fully addressed), “No” (item not addressed) or “No meta-analysis conducted.” Quality of each SR was categorized into four levels as follows: high level (no or one non-critical weakness), moderate level (more than one non-critical weakness), low level (one critical flaw with or without non-critical weaknesses) and critically low level (more than one critical flaw with or without non-critical weaknesses). In order to quantify the methodological quality, for non-critical items, we assigned “1” point for “Yes,” “0.5” for “Partial Yes,” and “0” for “No” or “No meta-analysis conducted,” respectively (12). For critical items, the score was double. The total AMSTAR-2 score was 23 points.

Reporting quality was assessed according to the Preferred Reporting Items for Systematic Reviews and Meta-analyses (PRISMA) statement, with a checklist of 27 items (Supplementary Table 2) (13). Each item was judged as “Yes” for total compliance, “Partial” for partial compliance, “No” for non-compliance and “Cannot answer” for limited information. The total score was obtained by adding “1” point for “Yes,” “0.5” for “Partial,” “0” for “No” and “Cannot answer” (7). The total PRISMA score was 27 points.

Two reviewers (QZ and FL) independently evaluated the methodological and reporting quality of the included SRs. Any conflicts between them resolved by consensus.

### Data Analysis

We compared the general characteristics, methodological and reporting quality between registered and non-registered SRs. Frequencies and percentages were used for categorial variables, whereas medians and interquartile ranges were taken as continuous variables. We used the Fisher's exact-test to analyze the differences in categorial items, and a two-sample Wilcoxon rank-sum test or Student *t*-test to evaluate the differences between continuous items.

For methodological and reporting quality, we calculated the frequency of “Yes” for each item, as well as the odds ratio (OR) with 95% confidence intervals (95% CIs) and *P*-values to compare the differences between registered and non-registered SRs. We also summarized mean scores and standard deviations obtained using AMSTAR-2 and PRISMA for each SR, and calculated mean differences and 95% CIs to compare the methodological and reporting quality between registered and non-registered SRs.

We also used univariate and multivariate linear regression analyses to explore factors related to methodological or reporting quality. The variables in the models, which were defined a priori, included impact factor, country of the first author, registration status, intervention type, number of RCTs, number of included patients, statistical result. In addition, we conducted sensitivity analyses to evaluate the robustness of statistical results by excluding the items related to registration (Item 2 for AMSTAR-2 and Item 5 for PRISMA) (7).

All analyses were performed in Stata 14.0 (StataCorp, College Station, TX, USA; www.stata.com), with statistical significance done using two-sided test where *p* < 0.05.

## Results

### Search Results

**Figure 1:**
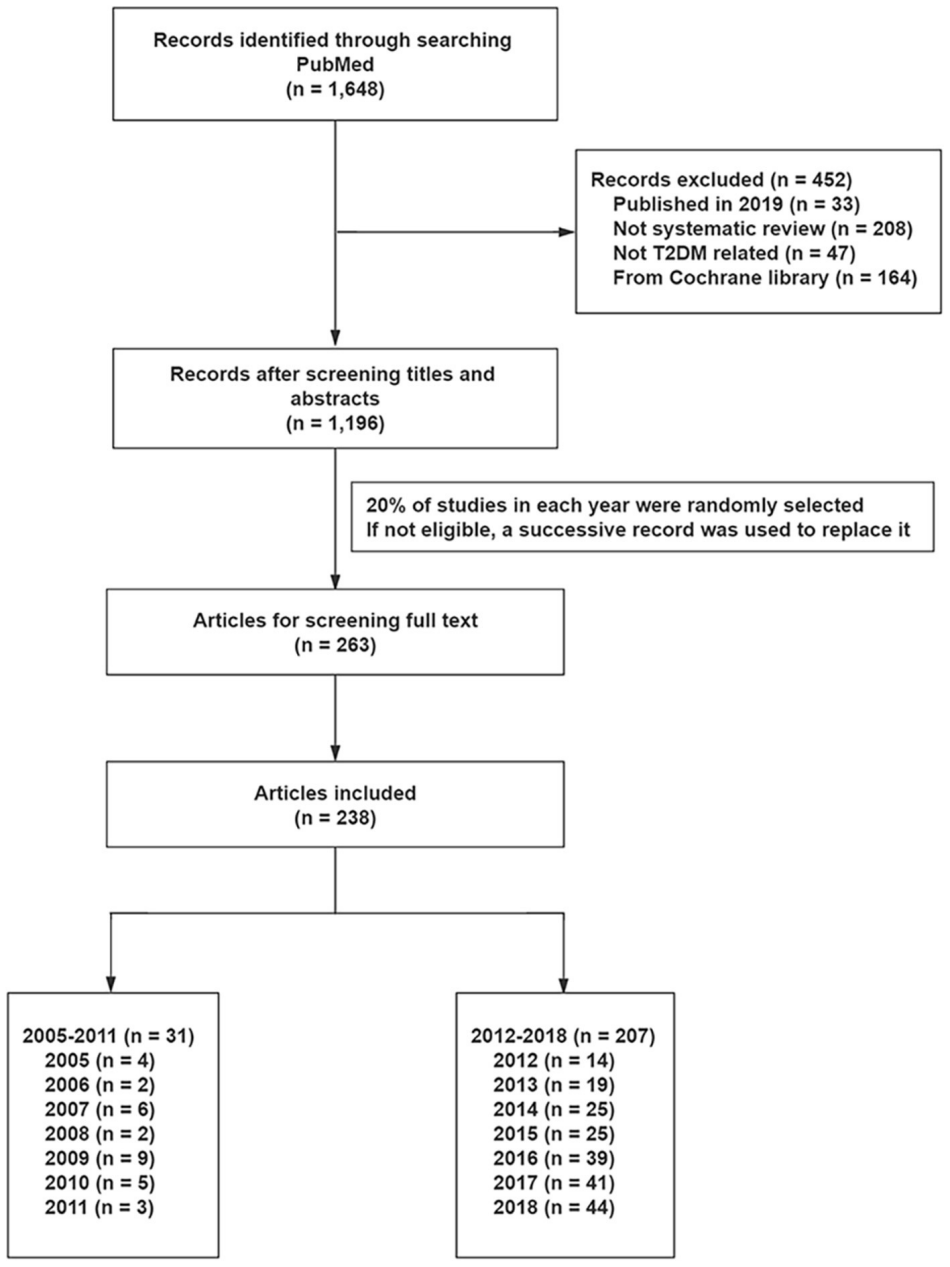
The flow diagram of literature selection. T2DM, type 2 diabetes mellitus.

We retrieved a total of 1,648 from the PubMed database, and found 1,196 to be eligible after reviewing titles and abstracts. Finally, 238 studies were randomly selected to evaluate methodological and reporting quality, including 31 SRs and 207 SRs before and after the introduction of PROSPERO, respectively (Figure 1 and Supplementary Table 3).

### Characteristics of the Selected Systematic Reviews

The general characteristics of SRs are outlined in Table 1. Among the selected studies, 33 were registered whereas 205 were non-registered SRs. Additionally, meta-analysis was conducted in 93.9% of the registered and 86.3% of the non-registered SRs. With regard to geographical origin, most of the included SRs were conducted in America/Canada (25.6%), Europe (28.6%), and China (28.2%). Twenty-one (63.6%) registered and 155 (75.6%) non-registered SRs were published in journals with an impact factor <5. In addition, the mean impact factor of registered SRs was 5, and 6% of the articles were published in journals with an impact factor over 10. The median number of RCTs and participants in the selected SRs was 12.0 (7.0, 22.0) and 3517.5 (1025.0, 13715.0), respectively. Furthermore, 57.6% of the included SRs evaluated the effect of pharmacological interventions, with 71.3% of included SRs exhibiting positive statistical results.

**Table 1:** Characteristics of included systematic reviews in study.

	**Total (*n* = 238)**	**Non-registered (*n* = 205)**	**Registered (*n* = 33)**	***P*-value**
Publication year (*n*, %)				0.011
2005–2011	31 (13.0)	31 (15.1)	0 (0.0)	
2012–2018	207 (87.0)	174 (84.9)	33 (100.0)	
Journal impact factor (*n*, %)				0.253
≦5	176 (73.9)	155 (75.6)	21 (63.6)	
5–10	44 (18.5)	34 (16.6)	10 (30.3)	
10–20	11 (4.6)	10 (4.9)	1 (3.0)	
>20	7 (2.9)	6 (2.9)	1 (3.0)	
Journal impact factor: median (IQR)	3.2 (2.4, 5.6)	3.2 (2.4, 4.8)	2.8 (2.4, 6.4)	0.766
Country or region (*n*, %)				0.503
USA/Canada	61 (25.6)	54 (26.3)	7 (21.2)	
Europe	68 (28.6)	58 (28.3)	10 (30.3)	
China	67 (28.2)	60 (29.3)	7 (21.2)	
Other Asian countries	24 (10.1)	19 (9.3)	5 (15.2)	
Others	18 (7.6)	14 (6.8)	4 (12.1)	
Meta-analysis (*n*, %)				0.393
Yes	208 (87.4)	177 (86.3)	31 (93.9)	
No	30 (12.6)	28 (13.7)	2 (6.1)	
No. of RCTs included: median (IQR)	12.0 (7.0, 22.0)	11.0 (7.0, 22.0)	13.0 (7.0, 24.0)	0.436
No. of patients included: median (IQR)	3517.5 (1025.0, 13715.0)	3783.5 (1062.0, 14356.5)	2229.5 (496.0, 9154.0)	0.172
Category of interventions (*n*, %)				0.368
Pharmacological	137 (57.6)	120 (58.5)	17 (51.5)	
Operation	4 (1.7)	3 (1.5)	1 (3.0)	
Psychological education	3 (1.3)	3 (1.5)	0 (0.0)	
Disease management	20 (8.4)	19 (9.3)	1 (3.0)	
Others	74 (31.1)	60 (29.3)	14 (42.4)	
Statistical result (*n*, %)				1.000
Positive^a^	169 (71.3)	145 (71.1)	24 (72.7)	
Negative^b^	68 (28.7)	59 (28.9)	9 (27.3)	

*IQR, interquartile range; RCTs, randomized controlled trials*.

*Positive^a^ means the result with statistical significance*.

*Negative^b^ means the result without statistical significance*.

### Trend of the Quality and Registered Rates

**Figure 2:**
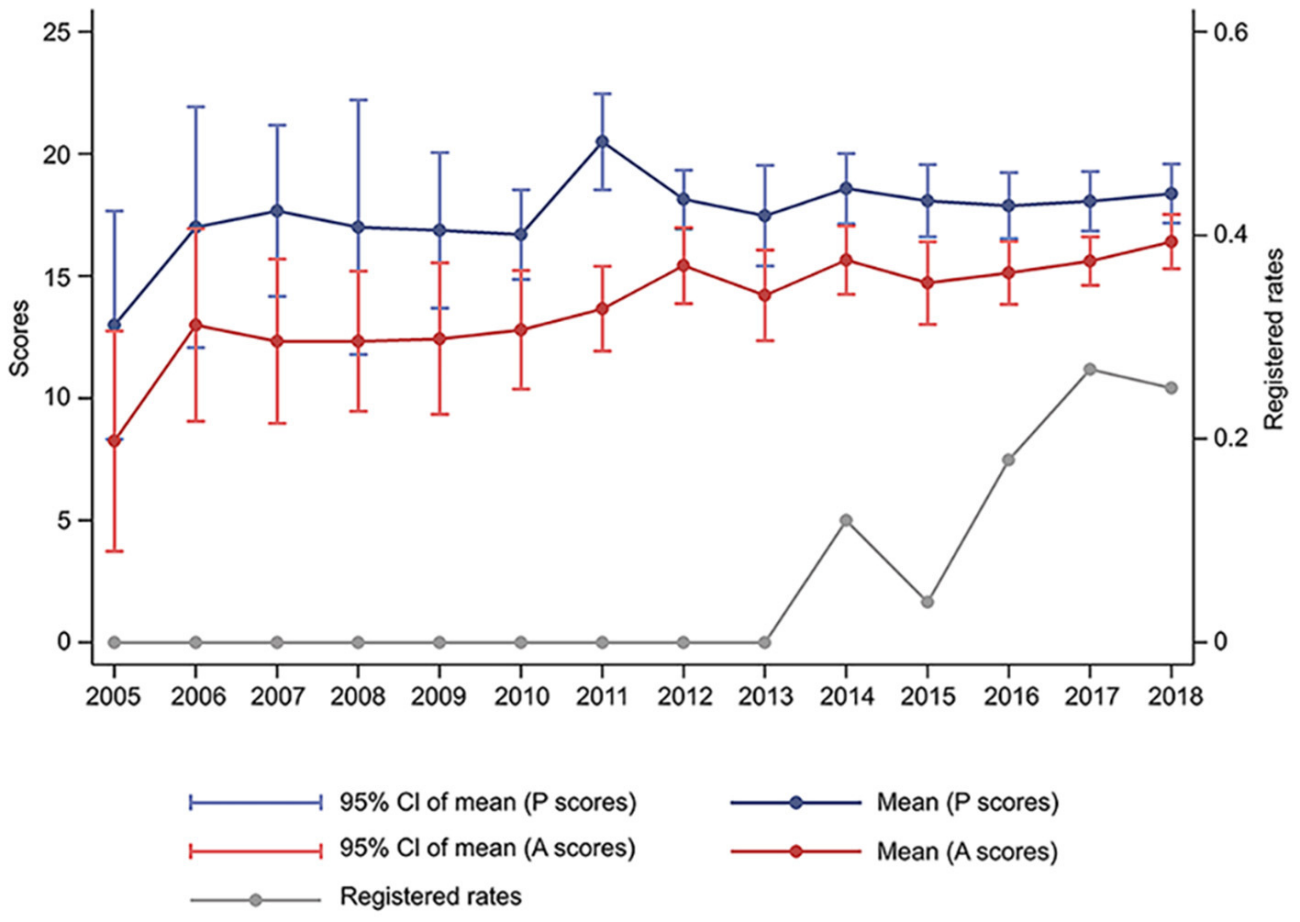
Changes of the quality and registered rates of systematic reviews in type 2 diabetes mellitus between 2005 and 2018. P, Preferred Reporting Items for Systematic Reviews and Meta-analyses; A, Assessment of Multiple Systematic Review 2.

The registered rates of SRs in T2DM increased from 0, in 2013, to about 25% in 2018. Methodological and reporting quality also improved steadily between 2005 and 2018. The mean of PRISMA scores were 13.0 and 18.4 points in 2005 and 2018, respectively, whereas those of AMSTAR-2 were 8.2 and 16.4 points in 2005 and 2018, respectively. According to the qualitative analysis of AMSTAR-2, most of the selected SRs had extremely low-level methodological quality, with 5 and 2 SRs exhibiting high and moderate levels, respectively (Figure 2 and Supplementary Figure 1).

### Methodological Quality of Included Systematic Reviews

**Figure 3:**
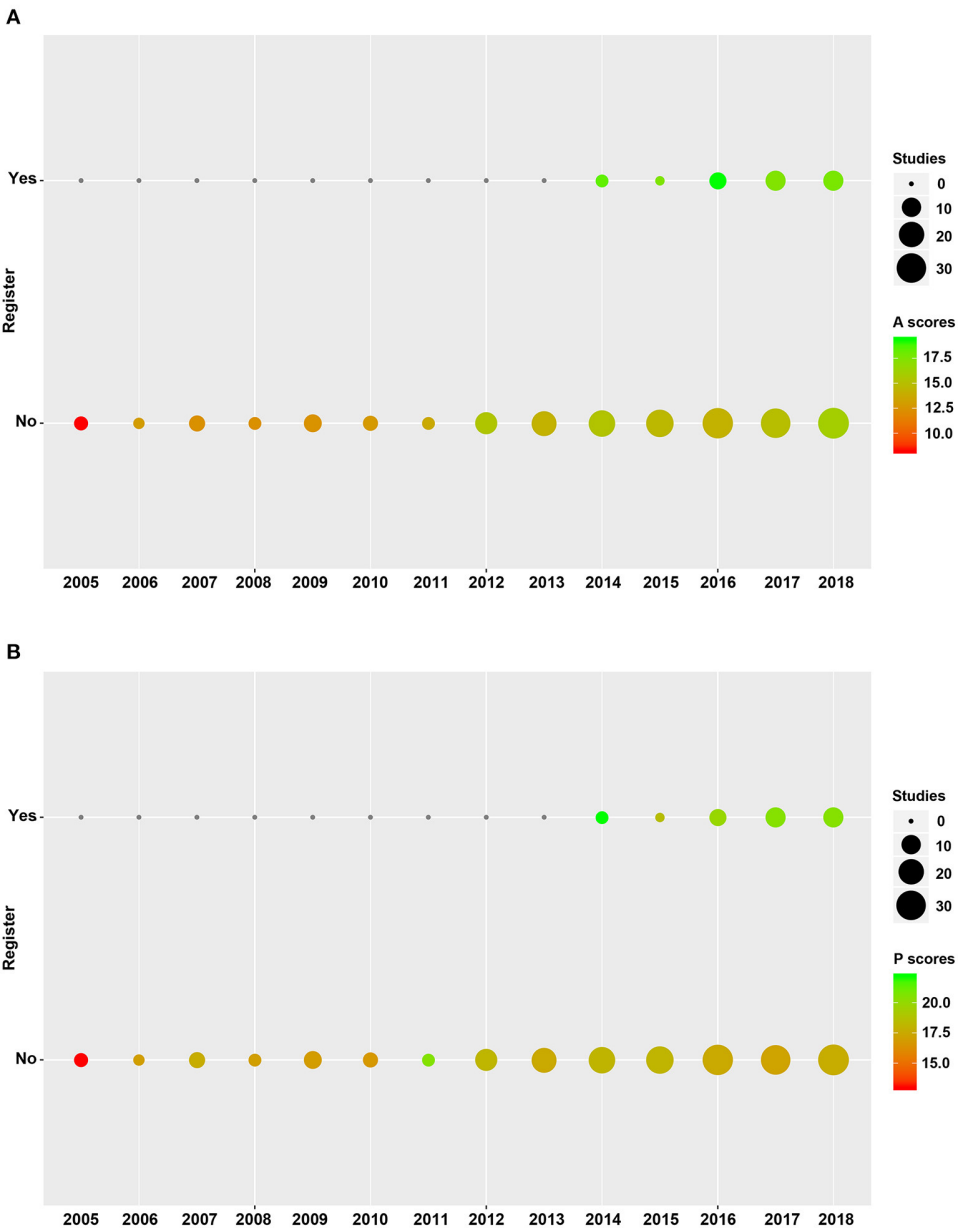
Quality of registered and non-registered systematic reviews included in the study. **(A)** Methodological quality of registered and non-registered systematic reviews. **(B)** Reporting quality between registered and non-registered systematic reviews. A, Assessment of Multiple Systematic Review 2; P, Preferred Reporting Items for Systematic Reviews and Meta-analyses.

**Figure 4:**
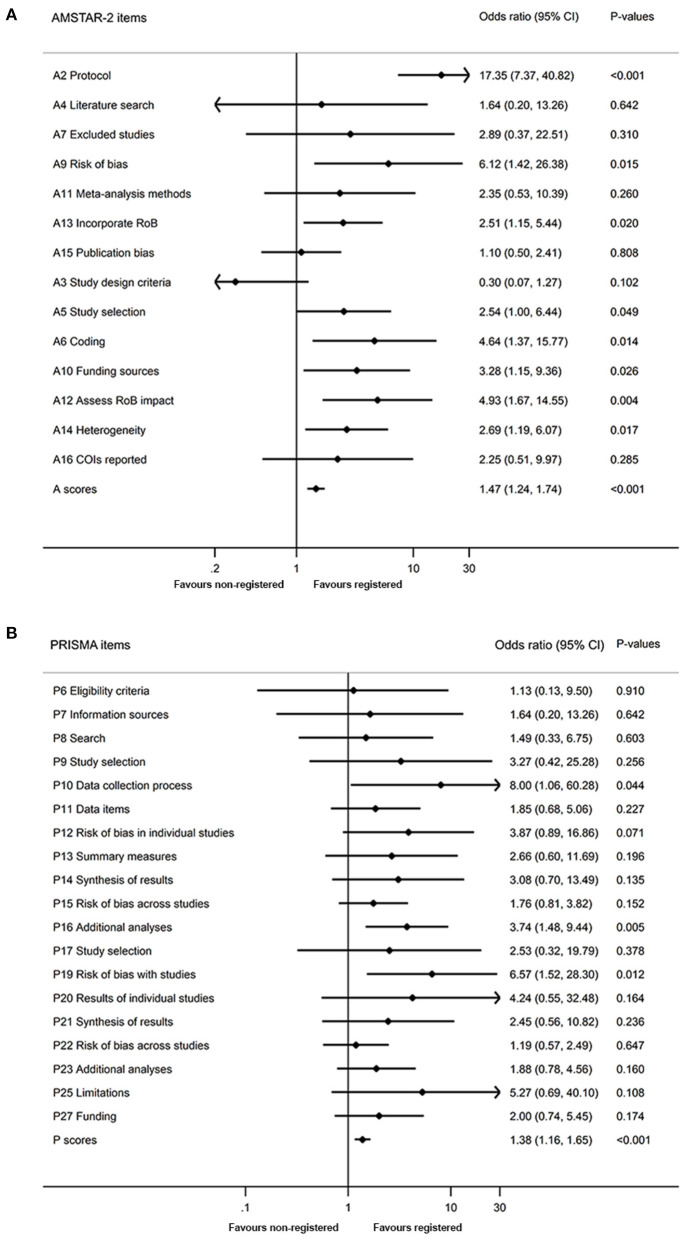
Comparison of quality between registered and non-registered reviews. **(A)** Comparison of methodological quality between registered and non-registered reviews. **(B)** Comparison of reporting quality between registered and non-registered reviews. A, Assessment of Multiple Systematic Review 2; P, Preferred Reporting Items for Systematic Reviews and Meta-analyses; RoB, risk of bias; COI, conflicts of interest.

Methodological quality of included SRs is shown in Figures 3A, 4A. We found higher mean AMSTAR-2 in registered than non-registered SRs (18.0 vs. 14.5, *P* = 0.000). Based on the AMSTAR-2 checklist, compliance rates of 8 items, including 3 critical ones (Item 2: Protocol, Item 9: Risk of bias, Item 13: Incorporate risk of bias), exhibited statistical significance between registered and non-registered SRs (*P* = 0.000, 0.048, 0.007, 0.005, 0.032, 0.002, 0.023, 0.015, in Item 2, 5, 6, 9, 10, 12, 13, 14, respectively). In the items describing publication bias and reporting funding sources of included studies, both registered and non-registered SRs had a low frequency (<40%) of “Yes” (Supplementary Table 4).

### Reporting Quality of Included Systematic Reviews

A summary of the reporting quality between registered and non-registered SRs is shown in Figures 3B, 4B. Results revealed a higher (20.4 ± 3.1) mean score of registered relative to non-registered (17.6, *P* = 0.000) SRs. Among the 5 items in PRISMA statement, we found significantly higher compliance rates in registered relative to non-registered SRs (Supplementary Table 5).

### Variables Associated With AMSTAR-2 Score and PRISMA Score

Univariate linear regression analyses showed that higher AMSTAR-2 scores were associated with registration status, country of the first author, and statistical results. However, only registration status and country of the first author were associated with AMSTAR-2 score based on multivariate regression analyses (Table 2). Similarly, PRISMA scores were related to registration status, country of the first author, and statistical results using both univariate and multivariate linear regression analyses (Table 2). Similar results were obtained after excluding items related to registration in sensitivity analyses (Supplementary Table 6).

**Table 2:** Results of linear regression analyses for variables associated with AMSTAR-2 scores and PRISMA scores.

**Variables**	**All items for AMSTAR-2**				**All items for PRISMA**			
	**Univariate**		**Multivariate**		**Univariate**		**Multivariate**	
	**Coef. (95% CI)**	***P***	**Coef. (95% CI)**	***P***	**Coef. (95% CI)**	***P***	**Coef. (95% CI)**	***P***
**Registered**								
No	0		0		0		0	
Yes	3.47 (2.10, 4.85)	0.000	3.39 (2.07, 4.71)	0.000	2.87 (1.46, 4.29)	0.000	2.87 (1.54, 4.20)	0.000
**Country or region**								
USA/Canada	0		0		0		0	
Europe	−0.04 (−1.34, 1.27)	0.958	−0.13 (−1.37, 1.11)	0.839	−0.16 (−1.48, 1.15)	0.806	−0.33 (−1.58, 0.92)	0.603
China	2.16 (0.85, 3.47)	0.001	2.04 (0.79, 3.29)	0.001	2.34 (1.02, 3.66)	0.001	2.06 (0.81, 3.32)	0.001
Other Asian countries	3.12 (1.34, 4.90)	0.001	2.74 (1.05, 4.43)	0.002	2.77 (0.97, 4.56)	0.003	2.37 (0.67, 4.07)	0.007
Others	1.49 (−0.50, 3.47)	0.141	1.05 (−0.83, 2.93)	0.273	0.45 (−1.55, 2.45)	0.660	−0.01 (−1.91, 1.88)	0.989
Journal impact factor	−0.03 (−0.10, 0.05)	0.464	—	–	0.06 (−0.01, 0.13)	0.110	—	–
No. of RCTs included	0.01 (−0.01, 0.02)	0.467	—	—	0.01 (−0.01, 0.02)	0.497	—	—
No. of patients included	0.00 (0.00, 0.00)	0.905	—	—	0.00 (0.00, 0.00)	0.172	—	—
**Interventions**								
Pharmacological	0		—		0		—	
Operation	0.77 (−3.16, 4.70)	0.700	—	—	1.49 (−2.48, 5.45)	0.462	—	—
Psychological education	−0.02 (−4.55, 4.50)	0.993	—	—	−0.68 (−5.25, 3.89)	0.769	—	—
Disease management	−0.08 (−1.93, 1.78)	0.933	—	—	−0.06 (−1.94, 1.81)	0.946	—	—
Others	0.48 (−0.64, 1.60)	0.402	—	—	−0.24 (−1.37, 0.88)	0.670	—	—
**Statistical result**								
Negative	0		0		0		0	
Positive	1.20 (0.10, 2.29)	0.032	0.90 (−0.11, 1.92)	0.081	2.14 (1.05, 3.23)	0.000	1.84 (0.82, 2.86)	0.000

*AMSTAR-2, Assessment of Multiple Systematic Review 2; PRISMA, Preferred Reporting Items for Systematic Reviews and Meta-analyses; Coef., coefficient; RCTs, randomized controlled trials*.

## Discussion

This is the first meta-epidemiological study investigating the quality of SRs for RCTs in T2DM as well as its association with the status of protocol registration. Our results indicated that methodological and reporting quality of SRs in T2DM improved over the last few years, with registered rates also increasing since 2013. Registered SRs exhibited better quality in study conduct and reporting relative to non-registered ones. However, both registered and non-registered SRs need to improve discussing publication bias as well as reporting funding sources.

Our results showed that the number of SRs describing T2DM raised from 20 in 2005 to 220 in 2018, with a 10-fold increase. Diabetes mellitus is thought to be associated with decreasing quality of life and increasing disability rates (14). Therefore, with the increasing incidence of T2DM (1), clinicians have paid more attention to the prognosis. In addition, more new antidiabetic drugs have been introduced over the past decades. In order to evaluate the effect and safety of those treatments, a large number of clinical trials have been conducted over the world, which may promote the increase of SRs in the field of T2DM (15–17). In the study, we also found that the quality of included SRs improved steadily over the recent years. This may be related to the establishment of PROSPERO and the development of the PRISMA statement. A prior registration can avoid overlapped reviews and help to make better clinical decisions (18). In addition, PRISMA statement can standardize many systematic reviews because more and more authors are required to provide PRISMA checklist during the submission process of SRs.

After the development of PROSPERO, registered rates of SRs for T2DM increased. However, only 25.0% of the SRs published in 2018 were registered in PROSPERO, consistent with previous studies. For example, <5.0% SRs had protocol registrations between 2010 and 2011 (19), and 8.5% dose-response mate-analyses completed registration between 2011 and 2015 (18). In the field of dentistry, Sideri et al. (20) reported that 20.3% of orthodontic non-Cochrane SRs were registered between 2012 and 2016, and Dos Santos et al. (21) found that 32.7% SRs reported protocol registrations in 2017. Among the SRs published in high-impact factor journals, 21.0% protocols were registered, with registered rates increasing from 5.6% in 2009 to 27.0% in 2015 (22). In the recent study, only 10.1% authors who conduct SRs completed all of their protocol registrations, while half of them never registered (23). These results confirm a universal pattern of low registered rates of SRs. It's reported that this phenomenon may be due to a lack of understanding of the importance of registration and the fear of idea being stolen (23). Therefore, more efforts are needed to promote protocol registration in the future.

In our study, there were no differences in the general characteristics between registered and non-registered SRs, except for the publication year. However, we found that most of the included studies had positive statistical results. Publication bias among clinical trials has been investigated in many researches (24–26), but there are few studies for non-Cochrane systematic reviews. In previous study, Moher et al. (2) found that publication bias might also exit among non-Cochrane SRs. Favorable significant findings were reported in 50.0% of non-Cochrane reviews and 14.4% of Cochrane reviews, respectively. In another research, 65.0% of authors who conducted SRs regarded statistically significant results as an important facilitator for publication, although statistical significance was not thought to be a main reason for not publishing SRs (27). More studies are needed to evaluate whether there is a publication bias among SRs based on statistical result.

A comparison between registered and non-registered SRs indicated, several aspects that need improvement. Firstly, reporting of study selection and data collection in non-registered SRs requires more details. Consequently, reviewers are required to complete the process in duplicate and contact authors of the included studies for more information (28). This may reduce the possibility of missing relevant studies and aid in avoiding bias and mistakes (29, 30). Secondly, despite data limitation, pre-specified additional analyses, such as sensitivity analysis, subgroup analysis or meta-regression still need to be conducted. These analyses can help in evaluating the robustness of results (28). Thirdly, authors need to assess, present and discuss risk of bias, including confounding, and sample selection bias, as well as bias when measuring exposures and outcomes, and selective reporting of outcomes and analyses for each included study (31). This will enable readers to realize the methodological shortcomings of relevant studies (28). Fourthly, non-registered review authors are encouraged to explain heterogeneity in their results. This is because study designs, analysis methods, population and interventions are considered important sources of heterogeneity (10), with proportion of statistical heterogeneity reported to affect decision making by clinicians (32). In addition, since the quality of non-registered SRs can be improved through connecting main findings to key groups, such as healthcare providers, users and policy makers (13), authors are encouraged to mention applicability of their findings to different groups and develop a standard way to evaluate applicability (33).

To date, registered and non-registered SRs have not adequately discussed publication bias and poorly reported funding sources for included studies. Adequate discussion of publication bias is important, as trials with positive results are more likely to be published (34). Despite a good understanding of this issue by a majority of authors, most of them do not adequately address it during research. For example, a previous study indicated that protocol registration may not prevent outcome publication bias (22). Researchers in the field of SRs should evaluate publication bias through deeper literature searches (10). SRs reporting T2DM have the lowest compliance rate in items reporting funding sources. Previous reports indicated that commercially-sponsored studies are unlikely to be published, since the resulting findings favor sponsors compared to those supported by other funding organizations (35–37). Therefore, it is important for reviewers to report funding sources of included studies.

There were some methodological biases in this study. Firstly, we only searched PubMed database to identify potential eligible SRs in T2DM. Although PubMed is the largest database of SRs currently available and it's most often used by clinicians worldwide, it doesn't completely cover published studies in the field of T2DM. It's undeniable that some SRs were only published in other electronic databases, which leads to a publication bias. Secondly, due to the limited time and resources, we randomly selected 20% of SRs in each year to assess the quality, instead of analyzing all eligible SRs retrieved from PubMed database. To our knowledge, the sample size used herein is the largest for evaluating the association between quality and registration status of non-Cochrane SRs. However, a possible selection bias cannot be ignored, which may result in the quality of SRs being overestimated or underestimated in our study. Researchers are encouraged to search more electronic databases and evaluate all eligible SRs to avoid methodological bias in future meta-epidemiological study.

In addition, the current study had other limitations. Firstly, we only included SRs published in English. It is possible that a different trend may be found using studies published in other languages. Secondly, derived the registration status of included SRs mainly based on related statements from published papers, which may lead to bias. For example, if registration status was not reported, we regarded the SR as non-registered. Thirdly, the assessment of methodological quality was dependent on author description, despite the fact that the actual process may be inconsistent with their description.

In conclusion, although methodological and reporting quality of SRs for RCTs in type 2 diabetes mellitus improved over the recent years, the overall quality remains low. Registered rates increased, and registered SRs exhibited better quality in study conduct and reporting compared to non-registered ones. Based on these findings, prospective registration, description of publication bias and reporting of funding sources are possible ways for improving the quality of systematic reviews. Future studies are expected to investigate more factors associated with methodological and reporting quality of SRs.

## Data Availability Statement

The raw data supporting the conclusions of this article will be made available by the authors, without undue reservation.

## Author Contributions

QZ, FL, YLiu, and HX had the idea and designed the study. HX and YLiu supervised the study, are the guarantors, had full access to all the data in the study, and had final responsibility for the decision to submit for publication. BL and JL did the statistical analysis. QZ, FL, and YLiu wrote the draft report and contributed to data acquisition. JX, SP, and YLi helped to organize the study. All authors contributed to the analysis, interpretation of data, revised the report, and approved the final version before submission.

## Conflict of Interest

The authors declare that the research was conducted in the absence of any commercial or financial relationships that could be construed as a potential conflict of interest.
