# Supplementary material for: Association Between Prospective Registration and Quality of Systematic Reviews in Type 2 Diabetes Mellitus: A Meta-epidemiological Study
Source: Front Med (Lausanne). 2021 Jun 28;8:639652. doi: 10.3389/fmed.2021.639652 (PMC8273164; doi:10.3389/fmed.2021.639652)
Supplement: Supplementary file 1 [file Data_Sheet_1.pdf]

Supplementary Table 1. AMSTAR-2 Checklist

AMSTAR 2

1. Did the research questions and inclusion criteria for the review include the components of PICO?

For Yes:	Optional (recommended)	
☐ Population<br>☐ Intervention<br>☐ Comparator group<br>☐ Outcome	☐ Timeframe for follow-up	☐ Yes<br>☐ No

2. Did the report of the review contain an explicit statement that the review methods were established prior to the conduct of the review and did the report justify any significant deviations from the protocol?

For Partial Yes:	For Yes:	
The authors state that they had a written protocol or guide that included ALL the following:	As for partial yes, plus the protocol should be registered and should also have specified:	
☐ review question(s)<br>☐ a search strategy<br>☐ inclusion/exclusion criteria<br>☐ a risk of bias assessment	☐ a meta-analysis/synthesis plan, if appropriate, *and*<br>☐ a plan for investigating causes of heterogeneity<br>☐ justification for any deviations from the protocol	☐ Yes<br>☐ Partial Yes<br>☐ No

3. Did the review authors explain their selection of the study designs for inclusion in the review?

For Yes, the review should satisfy ONE of the following:	
☐ *Explanation for* including only RCTs<br>☐ OR *Explanation for* including only NRSI<br>☐ OR *Explanation for* including both RCTs and NRSI	☐ Yes<br>☐ No

4. Did the review authors use a comprehensive literature search strategy?

For Partial Yes (all the following):	For Yes, should also have (all the following):	
☐ searched at least 2 databases (relevant to research question)<br>☐ provided key word and/or search strategy<br>☐ justified publication restrictions (eg, language)	☐ searched the reference lists/bibliographies of included studies<br>☐ searched trial/study registries<br>☐ included/consulted content experts in the field<br>☐ where relevant, searched for grey literature<br>☐ conducted search within 24 months of completion of the review	☐ Yes<br>☐ Partial Yes<br>☐ No

5. Did the review authors perform study selection in duplicate?

For Yes, either ONE of the following:	
☐ at least two reviewers independently agreed on selection of eligible studies and achieved consensus on which studies to include<br>☐ OR two reviewers selected a sample of eligible studies and achieved good agreement (at least 80 per cent), with the remainder selected by one reviewer	☐ Yes<br>☐ No

6. Did the review authors perform data extraction in duplicate?

For Yes, either ONE of the following:	
☐ at least two reviewers achieved consensus on which data to extract	☐ Yes

from included studies<br>☐ OR two reviewers extracted data from a sample of eligible studies <u>and</u> achieved good agreement (at least 80 per cent), with the remainder extracted by one reviewer		☐ No

7. Did the review authors provide a list of excluded studies and justify the exclusions?

For Partial Yes:	For Yes, must also have:	
☐ provided a list of all potentially relevant studies that were read in full text form but excluded from the review	☐ Justified the exclusion from the review of each potentially relevant study	☐ Yes<br>☐ Partial Yes<br>☐ No

8. Did the review authors describe the included studies in adequate detail?

For Partial Yes (ALL the following):	For Yes, should also have ALL the following:	
☐ described populations<br>☐ described interventions<br>☐ described comparators<br>☐ described outcomes<br>☐ described research designs	☐ described population in detail<br>☐ described intervention and comparator in detail (including doses where relevant)<br>☐ described study's setting<br>☐ timeframe for follow-up	☐ Yes<br>☐ Partial Yes<br>☐ No

9. Did the review authors use a satisfactory technique for assessing the risk of bias (RoB) in individual studies that were included in the review?

**RCTs**<br>For Partial Yes, must have assessed RoB from<br>☐ unconcealed allocation, *and*<br>☐ lack of blinding of patients and assessors when assessing outcomes (unnecessary for objective outcomes such as all cause mortality)	For Yes, must also have assessed RoB from:<br>☐ allocation sequence that was not truly random, *and*<br>☐ selection of the reported result from among multiple measurements or analyses of a specified outcome	☐ Yes<br>☐ Partial Yes<br>☐ No<br>☐ Includes only NRSI
**NRSI**<br>For Partial Yes, must have assessed RoB:<br>☐ from confounding, *and*<br>☐ from selection bias	For Yes, must also have assessed RoB:<br>☐ methods used to ascertain exposures and outcomes, *and*<br>☐ selection of the reported result from among multiple measurements or analyses of a specified outcome	☐ Yes<br>☐ Partial Yes<br>☐ No<br>☐ Includes only RCTs

10. Did the review authors report on the sources of funding for the studies included in the review?

For Yes	
☐ Must have reported on the sources of funding for individual studies included in the review. Note: Reporting that the reviewers looked for this information but it was not reported by study authors also qualifies	☐ Yes<br>☐ No

11. If meta-analysis was performed did the review authors use appropriate methods for statistical combination of results?

**RCTs**<br>For Yes:<br>☐ The authors justified combining the data in a meta-analysis<br>☐ AND they used an appropriate weighted technique to combine study results and adjusted for heterogeneity if present	☐ Yes<br>☐ No<br>☐ No meta-analysis

☐ AND investigated the causes of any heterogeneity	conducted
**For NRSI**<br>For Yes:<br>☐ The authors justified combining the data in a meta-analysis<br>☐ AND they used an appropriate weighted technique to combine study results, adjusting for heterogeneity if present<br>☐ AND they statistically combined effect estimates from NRSI that were adjusted for confounding, rather than combining raw data, or justified combining raw data when adjusted effect estimates were not available<br>☐ AND they reported separate summary estimates for RCTs and NRSI separately when both were included in the review	☐ Yes<br>☐ No<br>☐ No meta-analysis conducted

12. If meta-analysis was performed, did the review authors assess the potential impact of RoB in individual studies on the results of the meta-analysis or other evidence synthesis?

For Yes:<br>☐ included only low risk of bias RCTs<br>☐ OR, if the pooled estimate was based on RCTs and/or NRSI at variable RoB, the authors performed analyses to investigate possible impact of RoB on summary estimates of effect	☐ Yes<br>☐ No<br>☐ No meta-analysis conducted

13. Did the review authors account for RoB in individual studies when interpreting/discussing the results of the review?

For Yes:<br>☐ included only low risk of bias RCTs<br>☐ OR, if RCTs with moderate or high RoB, or NRSI were included the review provided a discussion of the likely impact of RoB on the results	☐ Yes<br>☐ No

14. Did the review authors provide a satisfactory explanation for, and discussion of, any heterogeneity observed in the results of the review?

For Yes:<br>☐ There was no significant heterogeneity in the results<br>☐ OR if heterogeneity was present the authors performed an investigation of sources of any heterogeneity in the results and discussed the impact of this on the results of the review	☐ Yes<br>☐ No

15. If they performed quantitative synthesis did the review authors carry out an adequate investigation of publication bias (small study bias) and discuss its likely impact on the results of the review?

For Yes:<br>☐ performed graphical or statistical tests for publication bias and discussed the likelihood and magnitude of impact of publication bias	☐ Yes<br>☐ No<br>☐ No meta-analysis conducted

16. Did the review authors report any potential sources of conflict of interest, including any funding they received for conducting the review?

For Yes:<br>☐ The authors reported no competing interests OR<br>☐ The authors described their funding sources and how they managed potential conflicts of interest	☐ Yes<br>☐ No

PICO, specification of inclusion criteria including the population, intervention, comparison, outcome; NRSI, non-randomized studies of interventions; RoB, risk of bias.

Supplementary Table 2. PRISMA 2009 Checklist

Section/topic	#	Checklist item	Compliance
TITLE			
Title	1	Identify the report as a systematic review, meta-analysis, or both.	□ Yes<br>□ Partial<br>□ No/Can't answer
ABSTRACT			
Structured summary	2	Provide a structured summary including, as applicable: background; objectives; data sources; study eligibility criteria, participants, and interventions; study appraisal and synthesis methods; results; limitations; conclusions and implications of key findings; systematic review registration number.	□ Yes<br>□ Partial<br>□ No/Can't answer
INTRODUCTION			
Rationale	3	Describe the rationale for the review in the context of what is already known.	□ Yes<br>□ Partial<br>□ No/Can't answer
Objectives	4	Provide an explicit statement of questions being addressed with reference to participants, interventions, comparisons, outcomes, and study design (PICOS).	□ Yes<br>□ Partial<br>□ No/Can't answer
METHODS			
Protocol and registration	5	Indicate if a review protocol exists, if and where it can be accessed (e.g., Web address), and, if available, provide registration information including registration number.	□ Yes<br>□ Partial<br>□ No/Can't answer
Eligibility criteria	6	Specify study characteristics (e.g., PICOS, length of follow-up) and report characteristics (e.g., years considered, language, publication status) used as criteria for eligibility, giving rationale.	□ Yes<br>□ Partial<br>□ No/Can't answer
Information sources	7	Describe all information sources (e.g., databases with dates of coverage, contact with study authors to identify additional studies) in the search and date last searched.	□ Yes<br>□ Partial<br>□ No/Can't answer
Search	8	Present full electronic search strategy for at least one database, including any limits used, such that it could be repeated.	□ Yes<br>□ Partial<br>□ No/Can't answer
Study selection	9	State the process for selecting studies (i.e., screening, eligibility, included in systematic review, and, if	□ Yes<br>□ Partial

		applicable, included in the meta-analysis).	□ No/Can't answer
Data collection process	10	Describe method of data extraction from reports (e.g., piloted forms, independently, in duplicate) and any processes for obtaining and confirming data from investigators.	□ Yes<br>□ Partial<br>□ No/Can't answer
Data items	11	List and define all variables for which data were sought (e.g., PICOS, funding sources) and any assumptions and simplifications made.	□ Yes<br>□ Partial<br>□ No/Can't answer
Risk of bias in individual studies	12	Describe methods used for assessing risk of bias of individual studies (including specification of whether this was done at the study or outcome level), and how this information is to be used in any data synthesis.	□ Yes<br>□ Partial<br>□ No/Can't answer
Summary measures	13	State the principal summary measures (e.g., risk ratio, difference in means).	□ Yes<br>□ Partial<br>□ No/Can't answer
Synthesis of results	14	Describe the methods of handling data and combining results of studies, if done, including measures of consistency (e.g., I^2) for each meta-analysis.	□ Yes<br>□ Partial<br>□ No/Can't answer
Risk of bias across studies	15	Specify any assessment of risk of bias that may affect the cumulative evidence (e.g., publication bias, selective reporting within studies).	□ Yes<br>□ Partial<br>□ No/Can't answer
Additional analyses	16	Describe methods of additional analyses (e.g., sensitivity or subgroup analyses, meta-regression), if done, indicating which were pre-specified.	□ Yes<br>□ Partial<br>□ No/Can't answer
RESULTS			
Study selection	17	Give numbers of studies screened, assessed for eligibility, and included in the review, with reasons for exclusions at each stage, ideally with a flow diagram.	□ Yes<br>□ Partial<br>□ No/Can't answer
Study characteristics	18	For each study, present characteristics for which data were extracted (e.g., study size, PICOS, follow-up period) and provide the citations.	□ Yes<br>□ Partial<br>□ No/Can't answer
Risk of bias within studies	19	Present data on risk of bias of each study and, if available, any outcome level assessment (see item 12).	□ Yes<br>□ Partial<br>□ No/Can't answer
Results of individual studies	20	For all outcomes considered (benefits or harms), present, for each study: (a) simple summary data for each intervention group (b) effect estimates and confidence	□ Yes<br>□ Partial<br>□ No/Can't answer

		intervals, ideally with a forest plot.	
Synthesis of results	21	Present results of each meta-analysis done, including confidence intervals and measures of consistency.	□ Yes<br>□ Partial<br>□ No/Can't answer
Risk of bias across studies	22	Present results of any assessment of risk of bias across studies (see Item 15).	□ Yes<br>□ Partial<br>□ No/Can't answer
Additional analysis	23	Give results of additional analyses, if done (e.g., sensitivity or subgroup analyses, meta-regression [see Item 16]).	□ Yes<br>□ Partial<br>□ No/Can't answer
DISCUSSION			
Summary of evidence	24	Summarize the main findings including the strength of evidence for each main outcome; consider their relevance to key groups (e.g., healthcare providers, users, and policy makers).	□ Yes<br>□ Partial<br>□ No/Can't answer
Limitations	25	Discuss limitations at study and outcome level (e.g., risk of bias), and at review-level (e.g., incomplete retrieval of identified research, reporting bias).	□ Yes<br>□ Partial<br>□ No/Can't answer
Conclusions	26	Provide a general interpretation of the results in the context of other evidence, and implications for future research.	□ Yes<br>□ Partial<br>□ No/Can't answer
FUNDING			
Funding	27	Describe sources of funding for the systematic review and other support (e.g., supply of data); role of funders for the systematic review.	□ Yes<br>□ Partial<br>□ No/Can't answer

PICO, specification of inclusion criteria including the population, intervention, comparison, outcome and study design.

Supplementary Table 3. Summary of included studies

Title	Publication Year	Journal	First author	No. of RCTs	No. of participants
Self-monitoring of blood glucose as part of a multi-component therapy among non-insulin requiring type 2 diabetes patients: a meta-analysis (1966-2004)	2005	Curr Med Res Opin	Sarol JN	8	1307
Orlistat in responding obese type 2 diabetic patients: meta-analysis findings and cost-effectiveness as rationales for reimbursement in Sweden and Switzerland	2005	Int J Obes (Lond)	Ruof J	7	2479
Effect of sibutramine on weight management and metabolic control in type 2 diabetes: a meta-analysis of clinical studies	2005	Diabetes Care	Vettor R	8	1093
The impact of ACE inhibitors or angiotensin II type 1 receptor blockers on the development of new-onset type 2 diabetes	2005	Diabetes Care	Gillespie EL	11	66608
Self-monitoring of glucose in type 2 diabetes mellitus: a Bayesian meta-analysis of direct and indirect comparisons	2006	Curr Med Res Opin	Jansen JP	12	2160
Effects of oral magnesium supplementation on glycaemic control in Type 2 diabetes: a meta-analysis of randomized double-blind controlled trials	2006	Diabet. Med.	Song Y	9	370
Meta-analysis of the effects of n-3 polyunsaturated fatty acids on haematological and thrombogenic factors in type 2 diabetes	2007	Diabetologia	Hartweg J	23	1075
Pharmacological and lifestyle interventions to prevent or delay type 2 diabetes in people with impaired glucose tolerance: systematic review and meta-analysis	2007	BMJ	Gillies CL	21	13080
The development of new-onset type 2 diabetes associated with choosing a calcium channel blocker compared to a diuretic or beta-blocker	2007	Curr Med Res Opin	Kuti EL	6	99006
Congestive heart failure and cardiovascular death in patients with prediabetes and type 2 diabetes given thiazolidinediones: a meta-analysis of randomised clinical trials	2007	Lancet	Lago RM	7	20191
Understanding the Quality Chasm for Hypertension Control in diabetes: a structured review of "co-maneuvers" used in clinical trials	2007	J Am Board Fam Med	Naik AD	20	NA
Effects of aerobic exercise on lipids and lipoproteins in adults with type 2 diabetes: a meta-analysis	2007	Public Health	Kelley GA	7	220

of randomized-controlled trials					
Addition of thiazolidinedione or exenatide to oral agents in type 2 diabetes: a meta-analysis	2008	Ann Pharmacother	Pinelli NR	22	9325
Educational interventions for migrant South Asians with Type 2 diabetes: a systematic review	2008	Diabet. Med.	Khunti K	5	1004
Statin therapy and risk of developing type 2 diabetes: a meta-analysis	2009	Diabetes Care	Rajpathak SN	6	57593
The effect of plant sterols or stanols on lipid parameters in patients with type 2 diabetes: a meta-analysis	2009	Diabetes Res. Clin. Pract.	Baker WL	5	148
Optimal insulin regimens in type 2 diabetes mellitus: systematic review and meta-analyses	2009	Diabetologia	Lasserson DS	22	4379
Self-monitoring of blood glucose in non-insulin treated patients with type 2 diabetes: a systematic review and meta-analysis	2009	Curr Med Res Opin	Allemann S	15	3270
Effect of intensive control of glucose on cardiovascular outcomes and death in patients with diabetes mellitus: a meta-analysis of randomised controlled trials	2009	Lancet	Ray KK	5	33040
Risk for nocturnal hypoglycemia with biphasic insulin aspart 30 compared with biphasic human insulin 30 in adults with type 2 diabetes mellitus: a meta-analysis	2009	Clinical Therapeutics	Davidson JA	9	1674
The association between intensive glycemic control and vascular complications in type 2 diabetes mellitus: a meta-analysis	2009	Nutr Metab Cardiovasc Dis	Ma J	8	32710
Nateglinide versus repaglinide for type 2 diabetes mellitus in China	2009	Acta Diabetol	Li C	4	955
Long-term use of thiazolidinediones and fractures in type 2 diabetes: a meta-analysis	2009	CMAJ	Loke YK	10	13715
Yoga Practice for the Management of Type II Diabetes Mellitus in Adults: A systematic review	2010	Evid Based Complement Alternat Med	Aljasir B	5	362
Effects of intensive glucose control on incidence of cardiovascular events in patients with type 2 diabetes: a meta-analysis	2010	Ann. Med.	Zhang CY	7	34144
Fibrates in the prevention of cardiovascular disease in patients with type 2 diabetes mellitus--a pooled meta-analysis of randomized placebo-controlled clinical trials	2010	Int. J. Cardiol.	Saha SA	6	11590

Dipeptydil peptidase-4 inhibitors in type 2 diabetes: a meta-analysis of randomized clinical trials	2010	Nutr Metab Cardiovasc Dis	Monami M	41	17810
Mediating the effect of self-care management intervention in type 2 diabetes: a meta-analysis of 47 randomised controlled trials	2010	Patient Educ Couns	Minet L	47	7677
Intensive glycemic control has no impact on the risk of heart failure in type 2 diabetic patients: evidence from a 37,229 patient meta-analysis	2011	Am. Heart J.	Castagno D	8	37229
Dipeptidyl Peptidase-4 Inhibitors and bone fractures: a meta-analysis of randomized clinical trials	2011	Diabetes Care	Monami M	28	21055
Effects and patient compliance of sustained-release versus immediate-release glipizides in patients with type 2 diabetes mellitus: a systematic review and meta-analysis	2011	J Evid Based Med	Wang L	19	1440
Changing physical activity behavior in type 2 diabetes: a systematic review and meta-analysis of behavioral interventions	2012	Diabetes Care	Avery L	17	1975
Clinical effect of metformin in children and adolescents with type 2 diabetes mellitus: a systematic review and meta-analysis	2012	J Family Community Med	Al-Shareef MA	2	347
Reappraisal of metformin efficacy in the treatment of type 2 diabetes: a meta-analysis of randomised controlled trials	2012	PLoS Med.	Boussageon R	13	13110
Berberine in the treatment of type 2 diabetes mellitus: a systemic review and meta-analysis	2012	Evid Based Complement Alternat Med	Dong H	14	1068
Thiazolidinedione use and cancer incidence in type 2 diabetes: a systematic review and meta-analysis	2012	Diabetes Metab.	Colmers IN	16	14422
Cinnamon in glycaemic control: Systematic review and meta analysis	2012	Clin Nutr	Akilen R	6	435
Effect of antidiabetic agents added to metformin on glycaemic control, hypoglycaemia and weight change in patients with type 2 diabetes: a network meta-analysis	2012	Diabetes Obes Metab	Liu SC	39	17860
Cardiovascular safety and glycemic control of glucagon-like peptide-1 receptor agonists for type 2 diabetes mellitus: a pairwise and network meta-analysis	2012	Diabetes Res. Clin. Pract.	Sun F	45	15883
Reno-protective effects of renin–angiotensin system blockade in type 2 diabetic patients: a systematic review and network meta-analysis	2012	Diabetologia	Vejakama P	28	134912

Choice of therapy in patients with type 2 diabetes inadequately controlled with metformin and a sulphonylurea: a systematic review and mixed-treatment comparison meta-analysis	2012	Open Med	McIntosh B	21	6610
Basal Supplementation of Insulin lispro protamine suspension versus insulin glargine and detemir for type 2 diabetes: meta-analysis of randomized controlled trials	2012	Diabetes Care	Esposito K	4	1336
Proportion of patients at HbA1c target <7% with eight classes of antidiabetic drugs in type 2 diabetes: systematic review of 218 randomized controlled trials with 78 945 patients	2012	Diabetes Obes Metab	Esposito K	218	78945
Meta-analysis of individual patient data in randomised trials of self monitoring of blood glucose in people with non-insulin treated type 2 diabetes	2012	BMJ	Farmer AJ	6	2552
The effect of linagliptin on glycaemic control and tolerability in patients with type 2 diabetes mellitus: a systematic review and meta-analysis	2012	Diabetes Obes Metab	Singh-Franco D	9	4246
Effectiveness of periodontal treatment to improve metabolic control in patients with chronic periodontitis and type 2 diabetes: a meta-analysis of randomized clinical trials.	2013	J. Periodontol.	Sgolastra F	5	315
Effects of exercise training on arterial function in type 2 diabetes mellitus: a systematic review and meta-analysis.	2013	Sports Med	Montero D	5	217
Effect of calcium channel blockers on incidence of diabetes: a meta-analysis.	2013	Effect of calcium channel blockers on incidence	Noto H	10	108118
Effect of short-term administration of cinnamon on blood pressure in patients with prediabetes and type 2 diabetes.	2013	Nutrition	Akilen R	3	139
Lifestyle interventions for patients with and at risk for type 2 diabetes: a systematic review and meta-analysis.	2013	Ann. Intern. Med.	Schellenberg ES	20	4200
Comparative efficacy of glimepiride and metformin in monotherapy of type 2 diabetes mellitus: meta-analysis of randomized controlled trials.	2013	Diabetol Metab Syndr	Zhu H	15	1681
Insulin degludec improves health-related quality of life (SF-36®) compared with insulin glargine in people with Type 2 diabetes starting on basal insulin: a meta-analysis of phase 3a trials.	2013	Diabet. Med.	Freemantle N	3	1922
Effects of high-protein diets on body weight, glycaemic control, blood lipids and blood pressure in	2013	Br. J. Nutr.	Dong JY	9	418

type 2 diabetes: meta-analysis of randomised controlled trials.					
Differences in the glucose-lowering efficacy of dipeptidyl peptidase-4 inhibitors between Asians and non-Asians: a systematic review and meta-analysis.	2013	Diabetologia	Kim YG	55	18328
Sulfonylureas and risk of falls and fractures: a systematic review.	2013	Drugs Aging	Lapane KL	9	16293
Intensive versus conventional glycemic control: what is best for patients with type 2 diabetes?	2013	Diabetes Metab Syndr	Ahmed AA	3	23182
Cardiovascular safety of sulfonylureas: a meta-analysis of randomized clinical trials.	2013	Diabetes Obes Metab	Monami M	115	45488
Effect of tight blood glucose control versus conventional control in patients with type 2 diabetes mellitus: a systematic review with meta-analysis of randomized controlled trials.	2013	Cardiovasc Ther	Buehler AM	6	27654
Insulin vs GLP-1 analogues in poorly controlled Type 2 diabetic subjects on oral therapy: a meta-analysis.	2013	J. Endocrinol. Invest.	Abdul-Ghani MA	7	2199
Blood pressure-lowering effects of GLP-1 receptor agonists exenatide and liraglutide: a meta-analysis of clinical trials.	2013	Diabetes Obes Metab	Wang B	16	5860
Exercise lowers postprandial glucose but not fasting glucose in type 2 diabetes: a meta-analysis of studies using continuous glucose monitoring.	2013	Diabetes Metab. Res. Rev.	MacLeod SF	8	116
Effects of exenatide and liraglutide on heart rate, blood pressure and body weight: systematic review and meta-analysis.	2013	BMJ Open	Robinson LE	32	NA
Volume of supervised exercise training impacts glycaemic control in patients with type 2 diabetes: a systematic review with meta-regression analysis.	2013	Diabetologia	Umpierre D	26	2253
Dipeptidyl peptidase-4 inhibitors and cardiovascular risk: a meta-analysis of randomized clinical trials.	2013	Diabetes Obes Metab	Monami M	70	41959
Assessment of the relative effectiveness and tolerability of treatments of type 2 diabetes mellitus: a network meta-analysis.	2014	Clin Ther	Zintzaras E	277	NA
Effect of GLP-1 mimetics on blood pressure and relationship to weight loss and glycemia lowering: results of a systematic meta-analysis and meta-regression.	2014	Am. J. Hypertens.	Katout M	33	12469
Diabetes and weight in comparative studies of bariatric surgery vs conventional medical therapy: a systematic review and meta-analysis.	2014	Obes Surg	Ribaric G	5	470

Effects of glucagon-like peptide-1 receptor agonists on cardiovascular risk: a meta-analysis of randomized clinical trials.	2014	Diabetes Obes Metab	Monami M	37	15398
Type 2 diabetes continuing medical education for general practitioners: what works? A systematic review.	2014	DIABETIC Medicine	Thepwongsa I	10	NA
Comparison of repaglinide and metformin versus metformin alone for type 2 diabetes: a meta-analysis of randomized controlled trials.	2014	Diabetes Res. Clin. Pract.	Yin J	22	2055
Clinical review: Effect of vitamin D3 supplementation on improving glucose homeostasis and preventing diabetes: a systematic review and meta-analysis.	2014	JCEM	Seida JC	35	43407
A nomogram to estimate the proportion of patients at hemoglobin A1c target <7% with noninsulin antidiabetic drugs in type 2 diabetes: a systematic review of 137 randomized controlled trials with 39,845 patients.	2014	Acta Diabetol	Esposito K	137	39845
The efficacy of dapagliflozin combined with hypoglycaemic drugs in treating type 2 diabetes mellitus: meta-analysis of randomised controlled trials.	2014	BMJ Open	Sun YN	12	6000
Resistance exercise versus aerobic exercise for type 2 diabetes: a systematic review and meta-analysis.	2014	Sports Med	Yang Z	12	626
Health education via mobile text messaging for glycemic control in adults with type 2 diabetes: a systematic review and meta-analysis.	2014	Prim Care Diabetes	Saffari M	10	960
Lixisenatide treatment for older patients with type 2 diabetes mellitus uncontrolled on oral antidiabetics: meta-analysis of five randomized controlled trials.	2014	Adv Ther	Hanefeld M	5	501
A meta-analysis of the hypoglycaemic risk in randomized controlled trials with sulphonylureas in patients with type 2 diabetes.	2014	Diabetes Obes Metab	Monami M	91	43404
A comparison of biphasic insulin aspart and insulin glargine administered with oral antidiabetic drugs in type 2 diabetes mellitus--a systematic review and meta-analysis.	2014	Int. J. Clin. Pract.	Rys P	5	1758
Effects of green tea or green tea extract on insulin sensitivity and glycaemic control in populations at risk of type 2 diabetes mellitus: a systematic review and meta-analysis of randomised controlled trials.	2014	J Hum Nutr Diet	Wang X	7	510

Metabolic effects of testosterone replacement therapy on hypogonadal men with type 2 diabetes mellitus: a systematic review and meta-analysis of randomized controlled trials.	2014	Asian J. Androl.	Cai X	5	351
Dapagliflozin compared with other oral anti-diabetes treatments when added to metformin monotherapy: a systematic review and network meta-analysis.	2014	Diabetes Obes Metab	Goring S	8	7861
Risk of diarrhea in patients with type 2 diabetes mellitus treated with sitagliptin: a meta-analysis of 30 randomized clinical trials.	2014	Drug Des Devel Ther	Zhao Q	30	8891
Computer-based interventions to improve self-management in adults with type 2 diabetes: a systematic review and meta-analysis.	2014	Diabetes Care	Pal K	16	3578
Effects of lixisenatide on elevated liver transaminases: systematic review with individual patient data meta-analysis of randomised controlled trials on patients with type 2 diabetes.	2014	BMJ Open	Gluud LL	15	NA
Combinational therapy with metformin and sodium-glucose cotransporter inhibitors in management of type 2 diabetes: systematic review and meta-analyses.	2014	Diabetes Res. Clin. Pract.	Zhang Q	7	3769
Comparison of GLP-1 analogues versus sitagliptin in the management of type 2 diabetes: systematic review and meta-analysis of head-to-head studies.	2014	PLoS ONE	Wang T	4	1755
Improving the adherence of type 2 diabetes mellitus patients with pharmacy care: a systematic review of randomized controlled trials.	2014	BMC Endocr Disord	Antoine SL	6	1025
Effect of tree nuts on glycemic control in diabetes: a systematic review and meta-analysis of randomized controlled dietary trials.	2014	PLoS ONE	Viguiliouk E	12	450
Effects of exercise on C-reactive protein, inflammatory cytokine and adipokine in patients with type 2 diabetes: a meta-analysis of randomized controlled trials.	2014	Metab. Clin. Exp.	Hayashino Y	14	824
Effect of GLP-1 receptor agonists on waist circumference among type 2 diabetes patients: a systematic review and network meta-analysis.	2015	Endocrine	Sun F	17	4365
Was there really any evidence that rosiglitazone increased the risk of myocardial infarction or death from cardiovascular causes?	2015	Pharmacoepidemiol Drug Saf	Stone JC	4	14291
Comparative efficacy and safety of antidiabetic drug regimens added to metformin monotherapy in patients with type 2 diabetes: a network meta-analysis.	2015	PLoS ONE	Mearns ES	62	32185

Effect of orlistat on glycaemic control in overweight and obese patients with type 2 diabetes mellitus: a systematic review and meta-analysis of randomized controlled trials.	2015	Obes Rev	Aldekhail NM	12	2802
Efficacy and safety of once-weekly glucagon-like peptide 1 receptor agonists for the management of type 2 diabetes: a systematic review and meta-analysis of randomized controlled trials.	2015	Diabetes Obes Metab	Karagiannis T	33	16003
Cardiovascular safety of the glucagon-like peptide-1 receptor agonist taspoglutide in people with type 2 diabetes: an individual participant data meta-analysis of randomized controlled trials.	2015	Diabetes Obes Metab	Seshasai SR	9	7056
Effect of Intensive Versus Standard Blood Glucose Control in Patients With Type 2 Diabetes Mellitus in Different Regions of the World: Systematic Review and Meta-analysis of Randomized Controlled Trials.	2015	J Am Heart Assoc	Sardar P	17	34967
Comparisons of the efficacy of glucose control, lipid profile, and β-cell function between DPP-4 inhibitors and AGI treatment in type 2 diabetes patients: a meta-analysis.	2015	Endocrine	Cai X	9	1975
The Effect of Glucagon-Like Peptide 1 Receptor Agonists on Weight Loss in Type 2 Diabetes: A Systematic Review and Mixed Treatment Comparison Meta-Analysis.	2015	PLoS ONE	Potts JE	27	9920
Sex-specific differences in diabetes prevention: a systematic review and meta-analysis.	2015	Diabetologia	Glechner A	12	13199
Psychological Interventions for the Management of Glycemic and Psychological Outcomes of Type 2 Diabetes Mellitus in China: A Systematic Review and Meta-Analyses of Randomized Controlled Trials.	2015	Front Public Health	Chapman A	45	5679
Effect of vitamins C and E on insulin resistance in diabetes: a meta-analysis study.	2015	Eur. J. Clin. Invest.	Khodaeian M	14	735
Lifestyle weight-loss intervention outcomes in overweight and obese adults with type 2 diabetes: a systematic review and meta-analysis of randomized clinical trials.	2015	J Acad Nutr Diet	Franz MJ	11	6754
Management of endocrine disease. Effects of telecare intervention on glycemic control in type 2 diabetes: a systematic review and meta-analysis of randomized controlled trials.	2015	Eur. J. Endocrinol.	Huang Z	18	3798
A systematic review and meta-analysis of tai chi for treating type 2 diabetes.	2015	Maturitas	Lee MS	15	754
Is HbA1c a valid surrogate for macrovascular and microvascular complications in type 2 diabetes?	2015	Diabetes Metab.	Bejan-Angoulvant T	8	33396
Biphasic vs basal bolus insulin regimen in Type 2 diabetes: a systematic review and meta-analysis of	2015	Diabet. Med.	Wang C	15	4384

randomized controlled trials.					
Psyllium fiber improves glycemic control proportional to loss of glycemic control: a meta-analysis of data in euglycemic subjects, patients at risk of type 2 diabetes mellitus, and patients being treated for type 2 diabetes mellitus.	2015	Am. J. Clin. Nutr.	Gibb RD	35	3455
Glucose-lowering drugs or strategies and cardiovascular outcomes in patients with or at risk for type 2 diabetes: a meta-analysis of randomised controlled trials.	2015	Lancet Diabetes Endocrinol	Udell JA	14	95502
Effects of exercise training using resistance bands on glycaemic control and strength in type 2 diabetes mellitus: a meta-analysis of randomised controlled trials.	2015	Acta Diabetol	McGinley SK	7	179
Does diabetes self-management education in conjunction with primary care improve glycemic control in Hispanic patients? A systematic review and meta-analysis.	2015	Diabetes Educ	Ferguson S	13	2784
Systematic review and meta-analysis of efficacy and safety of combinational therapy with metformin and dipeptidyl peptidase-4 inhibitors.	2015	Saudi Pharm J	Alanazi AS	19	12180
Assessing the efficacy and safety of combined DPP-4 inhibitor and insulin treatment in patients with type 2 diabetes: a meta-analysis.	2015	Int J Clin Exp Pathol	Chen C	7	3384
Effects of Omega-3 Fatty Acid Supplementation on Glucose Control and Lipid Levels in Type 2 Diabetes: A Meta-Analysis.	2015	PLoS ONE	Chen C	20	1209
Adherence to a Mediterranean diet and risk of diabetes: a systematic review and meta-analysis.	2015	Public Health Nutr	Schwingshackl L	1	3541
What is the impact of n-3 PUFAs on inflammation markers in Type 2 diabetic mellitus populations?: a systematic review and meta-analysis of randomized controlled trials.	2016	Lipids Health Dis	Lin N	8	955
Meta-Analysis of Effects of Sodium-Glucose Cotransporter 2 Inhibitors on Cardiovascular Outcomes and All-Cause Mortality Among Patients With Type 2 Diabetes Mellitus.	2016	Am. J. Cardiol.	Tang H	37	28859
A systematic review of commercial weight loss programmes' effect on glycemic outcomes among overweight and obese adults with and without type 2 diabetes mellitus.	2016	Obes Rev	Chaudhry ZW	18	NA
Network meta-analysis accurately predicted the outcome of a subsequent randomised trial comparing once weekly dulaglutide 1.5 mg and once daily liraglutide 1.8 mg.	2016	Int. J. Clin. Pract.	Fahrbach JL	15	7135
Comparing the Clinical Outcomes between Drug Eluting Stents and Bare Metal Stents in Patients	2016	PLoS ONE	Bundhun PK	10	830

with Insulin-Treated Type 2 Diabetes Mellitus: A Systematic Review and Meta-Analysis of 10 Randomized Controlled Trials.					
Effect of aerobic exercise intensity on glycemic control in type 2 diabetes: a meta-analysis of head-to-head randomized trials.	2016	Acta Diabetol	Liubaoerjijin Y	8	235
The Effect of Regular Exercise on Insulin Sensitivity in Type 2 Diabetes Mellitus: A Systematic Review and Meta-Analysis.	2016	Diabetes Metab J	Way KL	16	479
Effect of phosphodiesterase-5 inhibitors on glycemic control in person with type 2 diabetes mellitus: A systematic review and meta-analysis.	2016	J Clin Transl Endocrinol	Poolsup N	4	198
The efficacy and safety of liraglutide added to metformin in patients with diabetes: a meta-analysis of randomized controlled trials.	2016	Sci Rep	Gu J	9	4757
Silymarin in Type 2 Diabetes Mellitus: A Systematic Review and Meta-Analysis of Randomized Controlled Trials.	2016	J Diabetes Res	Voroneanu L	5	270
Efficacy and safety of the addition of a dipeptidyl peptidase-4 inhibitor to insulin therapy in patients with type 2 diabetes: A systematic review and meta-analysis.	2016	Diabetes Res. Clin. Pract.	Kim YG	9	4464
Strategies to Make Ramadan Fasting Safer in Type 2 Diabetics: A Systematic Review and Network Meta-analysis of Randomized Controlled Trials and Observational Studies.	2016	Medicine (Baltimore)	Lee SW	16	5081
DPP-4 inhibitor therapy and bone fractures in people with Type 2 diabetes - A systematic review and meta-analysis.	2016	Diabetes Res. Clin. Pract.	Mamza J	51	36402
Benefits and Harms of Sodium-Glucose Co-Transporter 2 Inhibitors in Patients with Type 2 Diabetes: A Systematic Review and Meta-Analysis.	2016	PLoS ONE	Storgaard H	34	9154
Blood pressure lowering and stroke events in type 2 diabetes: A network meta-analysis of randomized controlled trials.	2016	Int. J. Cardiol.	Xie XX	28	96765
Effects of vitamin D on serum lipid profile in patients with type 2 diabetes: A meta-analysis of randomized controlled trials.	2016	Clin Nutr	Jafari T	17	1363
Motivational interviewing and outcomes in adults with type 2 diabetes: A systematic review.	2016	Patient Educ Couns	Ekong G	14	4289
Effects of dipeptidyl peptidase-4 inhibitors on blood pressure in patients with type 2 diabetes: A	2016	J. Hypertens.	Zhang X	15	5636

systematic review and meta-analysis.					
Risk of heart failure with dipeptidyl peptidase-4 inhibitors in patients with type 2 diabetes mellitus: A meta-analysis of randomized controlled trials.	2016	Int. J. Cardiol.	Kundu A	3	36543
Efficacy and acceptability of very low energy diets in overweight and obese people with Type 2 diabetes mellitus: a systematic review with meta-analyses.	2016	Diabet. Med.	Rehackova L	4	223
The effectiveness of regular leisure-time physical activities on long-term glycemic control in people with type 2 diabetes: A systematic review and meta-analysis.	2016	Diabetes Res. Clin. Pract.	Pai LW	18	915
Effects on All-cause Mortality and Cardiovascular Outcomes in Patients With Type 2 Diabetes by Comparing Insulin With Oral Hypoglycemic Agent Therapy: A Meta-analysis of Randomized Controlled Trials.	2016	Clin Ther	Li J	3	15971
The role of global and regional DNA methylation and histone modifications in glycemic traits and type 2 diabetes: A systematic review.	2016	Nutr Metab Cardiovasc Dis	Muka T	53	10823
Metabolic Effects of Monounsaturated Fatty Acid-Enriched Diets Compared With Carbohydrate or Polyunsaturated Fatty Acid-Enriched Diets in Patients With Type 2 Diabetes: A Systematic Review and Meta-analysis of Randomized Controlled Trials.	2016	Diabetes Care	Qian F	28	1504
Practice nurse involvement in the management of adults with type 2 diabetes mellitus attending a general practice: results from a systematic review.	2016	Int J Evid Based Healthc	Parker D	5	4362
DPP-4 inhibitors and risk of infections: a meta-analysis of randomized controlled trials.	2016	Diabetes Metab. Res. Rev.	Yang W	74	58065
Efficacy and safety of canagliflozin in patients with type 2 diabetes: A meta-analysis of randomized controlled trials.	2016	Medicine (Baltimore)	Xiong W	7	5215
Adverse effects of incretin-based therapies on major cardiovascular and arrhythmia events: meta-analysis of randomized trials.	2016	Diabetes Metab. Res. Rev.	Wang T	100	102933
Chromium supplements for glycemic control in type 2 diabetes: limited evidence of effectiveness.	2016	Nutr. Rev.	Costello RB	20	NA
A systematic review and meta-analysis of glycemic control for the prevention of diabetic foot syndrome.	2016	J. Vasc. Surg.	Hasan R	9	10897

The effects of whole body vibration in patients with type 2 diabetes: a systematic review and meta-analysis of randomized controlled trials.	2016	Braz J Phys Ther	Robinson CC	2	70
Effects of interventions promoting monitoring of medication use and brief messaging on medication adherence for people with Type 2 diabetes: a systematic review of randomized trials.	2016	Diabet. Med.	Farmer AJ	15	4714
Efficacy and safety of empagliflozin as add-on to metformin for type 2 diabetes: a systematic review and meta-analysis.	2016	Eur. J. Clin. Pharmacol.	Zhong X	7	4256
Are Everolimus-Eluting Stents Associated With Better Clinical Outcomes Compared to Other Drug-Eluting Stents in Patients With Type 2 Diabetes Mellitus?: A Systematic Review and Meta-Analysis.	2016	Medicine (Baltimore)	Bundhun PK	10	11981
Efficacy and safety of dulaglutide in patients with type 2 diabetes: a meta-analysis and systematic review.	2016	Sci Rep	Zhang L	12	7440
Tumour Risk with Once-Weekly Glucagon-Like Peptide-1 Receptor Agonists in Type 2 Diabetes Mellitus Patients: A Systematic Review.	2016	Clin Drug Investig	Guo X	26	16090
Impact of diabetes duration on achieved reductions in glycated haemoglobin, fasting plasma glucose and body weight with liraglutide treatment for up to 28 weeks: a meta-analysis of seven phase III trials.	2016	Diabetes Obes Metab	Seufert J	7	3222
Probiotics for the management of type 2 diabetes mellitus: A systematic review and meta-analysis.	2016	Diabetes Res. Clin. Pract.	Samah S	6	317
Efficacy, safety and impact on β-cell function of dipeptidyl peptidase-4 inhibitors plus metformin combination therapy in patients with type 2 diabetes and the difference between Asians and Caucasians: a meta-analysis.	2016	J. Endocrinol. Invest.	Gao W	27	10089
Effects of Self-directed Exercise Programmes on Individuals with Type 2 Diabetes Mellitus: A Systematic Review Evaluating Their Effect on HbA and Other Metabolic Outcomes, Physical Characteristics, Cardiorespiratory Fitness and Functional Outcomes.	2017	Sports Med	Byrne H	28	NA
Medical Nutrition Therapy and Weight Loss Questions for the Evidence Analysis Library Prevention of Type 2 Diabetes Project: Systematic Reviews.	2017	J Acad Nutr Diet	Raynor HA	11	4213

Cardiovascular Safety of Dipeptidyl-Peptidase IV Inhibitors: A Meta-Analysis of Placebo-Controlled Randomized Trials.	2017	Am J Cardiovasc Drugs	Elgendy IY	90	66730
Comparison between SGLT2 inhibitors and DPP4 inhibitors added to insulin therapy in type 2 diabetes: a systematic review with indirect comparison meta-analysis.	2017	Diabetes Metab. Res. Rev.	Min SH	14	6980
Efficacy and safety of DPP-4 inhibitors in patients with type 2 diabetes: Meta-analysis of placebo-controlled randomized clinical trials.	2017	Diabetes Metab.	Rehman MB	36	54664
A systematic review and meta-analysis of trials of social network interventions in type 2 diabetes.	2017	BMJ Open	Spencer-Bonilla G	19	2319
An evaluation of the effectiveness of self-management interventions for people with type 2 diabetes after an acute coronary syndrome: a systematic review.	2017	J Clin Nurs	Tanash MI	4	146
Impact of metformin on cardiovascular disease: a meta-analysis of randomised trials among people with type 2 diabetes.	2017	Diabetologia	Griffin SJ	13	2079
Does Gender Influence the Cardiovascular Benefits Observed with Sodium Glucose Co-Transporter-2 (SGLT-2) Inhibitors? A Meta-Regression Analysis.	2017	Cardiol Ther	Mahmoud AN	26	22256
Continuous positive airway pressure and diabetes risk in sleep apnea patients: A systemic review and meta-analysis.	2017	Eur. J. Intern. Med.	Chen L	4	346
Effectiveness of Interventions for Promoting Objectively Measured Physical Activity of Adults With Type 2 Diabetes: A Systematic Review.	2017	J Phys Act Health	Alothman S	15	1326
Treatment with high dose salicylates improves cardiometabolic parameters: Meta-analysis of randomized controlled trials.	2017	Metab. Clin. Exp.	Baye E	24	1591
Bile acid sequestrants for glycemic control in patients with type 2 diabetes: A systematic review with meta-analysis of randomized controlled trials.	2017	J. Diabetes Complicat.	Hansen M	17	2950
Antimicrobial photodynamic therapy adjuvant to non-surgical periodontal therapy in patients with diabetes mellitus: A meta-analysis.	2017	Photodiagnosis Photodyn Ther	Abduljabbar T	4	109
Roux-En-Y Gastric Bypass in Type 2 Diabetes Patients with Mild Obesity: a Systematic Review and Meta-analysis.	2017	Obes Surg	Cohen R	5	NA

Impact of Egg Consumption on Cardiovascular Risk Factors in Individuals with Type 2 Diabetes and at Risk for Developing Diabetes: A Systematic Review of Randomized Nutritional Intervention Studies.	2017	Can J Diabetes	Richard C	6	768
Identifying non-pharmacological risk factors for falling in older adults with type 2 diabetes mellitus: a systematic review.	2017	Disabil Rehabil	Gravesande J	2	108
Efficacy and effectiveness of screen and treat policies in prevention of type 2 diabetes: systematic review and meta-analysis of screening tests and interventions.	2017	BMJ	Barry E	50	NA
Efficacy of low carbohydrate diet for type 2 diabetes mellitus management: A systematic review and meta-analysis of randomized controlled trials.	2017	Diabetes Res. Clin. Pract.	Meng Y	9	734
The effects of dipeptidyl peptidase-4 inhibitors on bone fracture among patients with type 2 diabetes mellitus: A network meta-analysis of randomized controlled trials.	2017	PLoS ONE	Yang J	75	70207
A network meta-analysis for efficacy and safety of seven regimens in the treatment of type II diabetes.	2017	Biomed. Pharmacother.	Wang LG	8	3180
The effect of vitamin D supplementation on glucose metabolism in type 2 diabetes mellitus: A systematic review and meta-analysis of intervention studies.	2017	J. Diabetes Complicat.	Lee CJ	29	3324
How to prevent type 2 diabetes in women with previous gestational diabetes? A systematic review of behavioural interventions.	2017	Prim Care Diabetes	Pedersen ALW	10	3636
Psychosocial intervention for patients with type 2 diabetes mellitus and comorbid depression: a meta-analysis of randomized controlled trials.	2017	Neuropsychiatr Dis Treat	Xie J	31	2616
Incretin based treatments and mortality in patients with type 2 diabetes: systematic review and meta-analysis.	2017	BMJ	Liu J	189	155145
Comparative Effectiveness of Adding Alogliptin to Metformin Plus Sulfonylurea with Other DPP-4 Inhibitors in Type 2 Diabetes: A Systematic Review and Network Meta-Analysis.	2017	Diabetes Ther	Kay S	8	4109
Multiple effects of probiotics on different types of diabetes: a systematic review and meta-analysis of randomized, placebo-controlled trials.	2017	J. Pediatr. Endocrinol. Metab.	Wang X	18	1056
Effectiveness of chronic care models for the management of type 2 diabetes mellitus in Europe: a	2017	BMJ Open	Bongaerts BW	8	9529

systematic review and meta-analysis.					
Aerobic Interval Training and Cardiometabolic Health in Patients with Type 2 Diabetes: A Meta-Analysis.	2017	Front Physiol	Qiu S	7	189
A systematic review of the benefits and harms of dipeptidyl peptidase-4 inhibitor for chronic kidney disease.	2017	Hemodial Int	Kamiya H	8	1747
Long-term efficacy and safety of sodium-glucose cotransporter-2 inhibitors as add-on to metformin treatment in the management of type 2 diabetes mellitus: A meta-analysis.	2017	Medicine (Baltimore)	Li J	6	4536
Effect of Dipeptidyl Peptidase-4 Inhibitors on Heart Failure: A Network Meta-Analysis.	2017	Value Health	Guo WQ	50	NA
SCORE-IT (Selecting Core Outcomes for Randomised Effectiveness trials In Type 2 diabetes): a systematic review of registered trials.	2017	Trials	Harman NL	138	1444
Comparative effectiveness of glycemic control in patients with type 2 diabetes treated with GLP-1 receptor agonists: a network meta-analysis of placebo-controlled and active-comparator trials.	2017	Diabetes Metab Syndr Obes	Orme ME	29	18543
100 % Fruit juice and measures of glucose control and insulin sensitivity: a systematic review and meta-analysis of randomised controlled trials.	2017	J Nutr Sci	Murphy MM	18	960
Physical Exercise on Inflammatory Markers in Type 2 Diabetes Patients: A Systematic Review of Randomized Controlled Trials.	2017	Oxid Med Cell Longev	Melo LC	11	726
Glycemic Control During Continuous Subcutaneous Insulin Infusion Versus Multiple Daily Insulin Injections in Type 2 Diabetes: Individual Patient Data Meta-analysis and Meta-regression of Randomized Controlled Trials.	2017	Diabetes Care	Pickup JC	5	287
Effects of yoga in adults with type 2 diabetes mellitus: A meta-analysis.	2017	J Diabetes Investig	Cui J	12	864
SGLT2 inhibitors and risk of cancer in type 2 diabetes: a systematic review and meta-analysis of randomised controlled trials.	2017	Diabetologia	Tang H	46	34569
Prevention of Type 2 Diabetes in U.S. Hispanic Youth: A Systematic Review of Lifestyle Interventions.	2017	Am J Prev Med	McCurley JL	11	6384
A meta-analysis comparing clinical effects of short- or long-acting GLP-1 receptor agonists versus insulin treatment from head-to-head studies in type 2 diabetic patients.	2017	Diabetes Obes Metab	Abd El Aziz MS	19	8854

Effect of continuous positive airway pressure on glucose metabolism in adults with type 2 diabetes: a systematic review and meta-analysis of randomized controlled trials.	2018	Sleep Breath	Zhu B	6	496
SGLT-2 inhibitors and the risk of infections: a systematic review and meta-analysis of randomized controlled trials.	2018	Acta Diabetol	Puckrin R	86	50880
Semaglutide for type 2 diabetes mellitus: A systematic review and meta-analysis.	2018	Diabetes Obes Metab	Andreadis P	12	9501
Comparison of dipeptidyl peptidase-4 inhibitors and pioglitazone combination therapy versus pioglitazone monotherapy in type 2 diabetes: A system review and meta-analysis.	2018	Medicine (Baltimore)	Wang B	7	4335
Effectiveness of smartphone technologies on glycaemic control in patients with type 2 diabetes: systematic review with meta-analysis of 17 trials.	2018	Obes Rev	Wu IXY	17	2225
A systematic search and qualitative review of reporting bias of lifestyle interventions in randomized controlled trials of diabetes prevention and management.	2018	Nutr J	Riediger ND	19	2542
Dietary program and physical activity impact on biochemical markers in patients with type 2 diabetes: A systematic review.	2018	Aten Primaria	Barreira E	30	3494
The Effects of Qigong on Type 2 Diabetes Mellitus: A Systematic Review and Meta-Analysis.	2018	Evid Based Complement Alternat Med	Meng D	21	1326
Update in Cardiovascular Safety of Glucagon Like Peptide-1 Receptor Agonists in Patients With Type 2 Diabetes. A Mixed Treatment Comparison Meta-Analysis of Randomised Controlled Trials.	2018	Heart Lung Circ	Al Yami MS	4	33457
Statin therapy on glycemic control in type 2 diabetic patients: A network meta-analysis.	2018	J Clin Pharm Ther	Cui JY	23	2707
Effects of Structured Versus Unstructured Self-Monitoring of Blood Glucose on Glucose Control in Patients With Non-insulin-treated Type 2 Diabetes: A Meta-Analysis of Randomized Controlled Trials.	2018	J Diabetes Sci Technol	Mannucci E	11	2199
Blood glucose reduction by diabetic drugs with minimal hypoglycaemia risk for cardiovascular outcomes: Evidence from meta-regression analysis of randomized controlled trials.	2018	Diabetes Obes Metab	Huang CJ	10	92400
Addition of dipeptidyl peptidase-4 inhibitors to insulin treatment in type 2 diabetes patients: A meta-analysis.	2018	J Diabetes Investig	Yang W	36	13786

Exercise or physical activity and cognitive function in adults with type 2 diabetes, insulin resistance or impaired glucose tolerance: a systematic review.	2018	Eur Rev Aging Phys Act	Zhao RR	3	578
The effect of concomitant DPPIVi use on glycaemic control and hypoglycaemia with insulin glargine 300 U/mL (Gla-300) versus insulin glargine 100 U/mL (Gla-100) in people with type 2 diabetes: A patient-level meta-analysis of EDITION 2 and 3.	2018	PLoS ONE	Yale JF	2	1689
The effects of folate supplementation on glucose metabolism and risk of type 2 diabetes: a systematic review and meta-analysis of randomized controlled trials.	2018	Ann Epidemiol	Zhao JV	18	21081
Meta-analysis of metabolic surgery versus medical treatment for microvascular complications in patients with type 2 diabetes mellitus.	2018	Br J Surg	Billeter AT	3	309
Long-term risk of rosiglitazone on cardiovascular events - a systematic review and meta-analysis.	2018	Endokrynol Pol	Cheng D	11	20079
The Effects of Tai Chi on Type 2 Diabetes Mellitus: A Meta-Analysis.	2018	J Diabetes Res	Chao M	14	798
The Effects of Postprandial Exercise on Glucose Control in Individuals with Type 2 Diabetes: A Systematic Review.	2018	Sports Med	Borror A	12	135
Coronary artery bypass grafting versus percutaneous coronary intervention in patients with noninsulin treated type 2 diabetes mellitus: A meta-analysis of randomized controlled trials.	2018	Diabetes Metab. Res. Rev.	Wang Y	5	2270
Better glycaemic control and less hypoglycaemia with insulin glargine 300 U/mL vs glargine 100 U/mL: 1-year patient-level meta-analysis of the EDITION clinical studies in people with type 2 diabetes.	2018	Diabetes Obes Metab	Ritzel R	3	2496
Metformin, Asian ethnicity and risk of prostate cancer in type 2 diabetes: a systematic review and meta-analysis.	2018	BMC Cancer	Chen CB	2	NA
Impact of Exercise Training on Cardiac Function Among Patients With Type 2 Diabetes: A SYSTEMATIC REVIEW AND META-ANALYSIS.	2018	J Cardiopulm Rehabil Prev	Anand V	6	441
Tolerability and Efficacy of Ipragliflozin in The Management of Inadequately Controlled Type 2 Diabetes mellitus: A Systematic Review and Meta-analysis.	2018	Exp. Clin. Endocrinol. Diabetes	Elgebaly A	13	2535
Risks of diabetic foot syndrome and amputation associated with sodium glucose co-transporter 2	2018	Diabetes Metab.	Li D	14	26167

inhibitors: A Meta-analysis of Randomized Controlled Trials.					
Antioxidant effects of vitamins in type 2 diabetes: a meta-analysis of randomized controlled trials.	2018	Diabetol Metab Syndr	Balbi ME	30	1430
Probiotic supplementation for management of cardiovascular risk factors in adults with type II diabetes: A systematic review and meta-analysis.	2018	Clin Nutr	Hendijani F	11	641
The Effectiveness of Continuous Glucose Monitoring in Patients with Type 2 Diabetes: A Systematic Review of Literature and Meta-analysis.	2018	Diabetes Technol. Ther.	Park C	7	1384
Comparison of antidiabetic drugs added to sulfonylurea monotherapy in patients with type 2 diabetes mellitus: A network meta-analysis.	2018	PLoS ONE	Qian D	24	10032
Effects of pioglitazone treatment on blood leptin levels in patients with type 2 diabetes	2018	J Diabetes Investig	Satoshi Ida	10	578
Effects of Prebiotic and Synbiotic Supplementation on Glycaemia and Lipid Profile in Type 2 Diabetes: A Meta-Analysis of Randomized Controlled Trials.	2018	Adv Pharm Bull	Mahboobi S	10	506
Ethnic Differences in Efficacy and Safety of Alogliptin: A Systematic Review and Meta-Analysis.	2018	Diabetes Ther	Cai Y	15	4456
Chromium supplementation for adjuvant treatment of type 2 diabetes mellitus: Results from a pooled analysis.	2018	Mol Nutr Food Res	Huang H	28	1295
Effects of Omega-3 Supplementation on Adipocytokines in Prediabetes and Type 2 Diabetes Mellitus: Systematic Review and Meta-Analysis of Randomized Controlled Trials.	2018	Diabetes Metab J	Becic T	14	685
Efficacy of Pharmacist Based Diabetes Educational Interventions on Clinical Outcomes of Adults With Type 2 Diabetes Mellitus: A Network Meta-Analysis.	2018	Front Pharmacol	Bukhsh A	43	6259
The effect of diabetes self-management education on HbA1c and quality of life in African-Americans: a systematic review and meta-analysis.	2018	BMC Health Serv Res	Cunningham AT	12	2234
Quality improvement strategies at primary care level to reduce inequalities in diabetes care: an equity-oriented systematic review.	2018	BMC Endocr Disord	Terens N	58	17786
Association of Patient Profile with Glycemic Control and Hypoglycemia with Insulin Glargine 300 U/mL in Type 2 Diabetes: A Post Hoc Patient-Level Meta-Analysis.	2018	Diabetes Ther	Twigg SM	3	2496
Direct head-to-head comparison of glycaemic durability of dipeptidyl peptidase-4 inhibitors and	2018	Diabetes Obes Metab	Chen K	8	5987

sulphonylureas in patients with type 2 diabetes mellitus: A meta-analysis of long-term randomized controlled trials.					
Comparing SGLT-2 inhibitors to DPP-4 inhibitors as an add-on therapy to metformin in patients with type 2 diabetes: A systematic review and meta-analysis.	2018	Diabetes Metab.	Mishriky BM	7	2884
Metformin induces significant reduction of body weight, total cholesterol and LDL levels in the elderly - A meta-analysis.	2018	PLoS ONE	Solymár M	6	1541
The effect of n-3 PUFAs on circulating adiponectin and leptin in patients with type 2 diabetes mellitus: a systematic review and meta-analysis of randomized controlled trials.	2018	Acta Diabetol	Farimani AR	10	494
Cardiovascular Safety, Long-Term Noncardiovascular Safety, and Efficacy of Sodium-Glucose Cotransporter 2 Inhibitors in Patients With Type 2 Diabetes Mellitus: A Systemic Review and Meta-Analysis With Trial Sequential Analysis.	2018	J Am Heart Assoc	Zhang XL	5	352476

Supplementary Table 4. Details of AMSTAR-2 Assessment

AMSTAR-2 items	Nonregistered (n=205)	Registered (n=33)	*P*-value
Item 1. Did the research questions and inclusion criteria for the review include the components of PICO? (n, %)			1.000
Yes	202 (98.5)	33 (100.0)	
No	3 (1.5)	0 (0.0)	
Item 2. Did the report of the review contain an explicit statement that the review methods were established prior to the conduct of the review and did the report justify any significant deviations from the protocol? (n, %)			0.000
Yes	24 (11.7)	23 (69.7)	
Partial Yes	0 (0.0)	0 (0.0)	
No	181 (88.3)	10 (30.3)	
Item 3. Did the review authors explain their selection of the study designs for inclusion in the reviews? (n, %)			0.114
Yes	199 (97.1)	30 (90.9)	
No	6 (2.9)	3 (9.1)	
Item 4. Did the review authors use a comprehensive literature search strategy? (n, %)			0.896
Yes	181 (88.3)	31 (93.9)	
Partial Yes	14 (6.8)	1 (3.0)	
No	10 (4.9)	1 (3.0)	
Item 5. Did the review authors perform study selection in duplicate? (n, %)			0.048
Yes	131 (63.9)	27 (81.8)	
No	74 (36.1)	6 (18.2)	
Item 6. Did the review authors perform data extraction in duplicate? (n, %)			0.007
Yes	140 (68.3)	30 (90.9)	
No	65 (31.7)	3 (9.1)	
Item 7. Did the review authors provide a list of excluded studies and justify the exclusions? (n, %)			0.669
Yes	185 (90.2)	32 (97.0)	
Partial Yes	3 (1.5)	0 (0.0)	
No	17 (8.3)	1 (3.0)	
Item 8. Did the review authors describe the included studies in adequate detail? (n, %)			0.145
Yes	100 (48.8)	11 (33.3)	
Partial Yes	98 (47.8)	22 (66.7)	
No	7 (3.4)	0 (0.0)	
Item 9. Did the review authors use a satisfactory technique for assessing the risk of bias (RoB) in individual that were included in the review? (n, %)			0.005
Yes	131 (63.9)	30 (90.9)	
Partial Yes	16 (7.8)	1 (3.0)	

No	58 (28.3)	2 (6.1)	
Item 10. Did the review authors report on the sources of funding for the studies included in the review? (n, %)			0.032
Yes	13 (6.3)	6 (18.2)	
No	192 (93.7)	27 (81.8)	
Item 11. If meta-analysis was performed did the review authors use appropriate methods for statistical combination of results? (n, %)			0.389
Yes	178 (86.8)	31 (93.9)	
No/ No meta-analysis conducted	27 (13.2)	2 (6.1)	
Item 12. If meta-analysis was performed, did the review authors assess the potential impact of RoB in individual studies on the results of the meta-analysis or other evidence synthesis? (n, %)			0.002
Yes	122 (59.5)	29 (87.9)	
No/ No meta-analysis conducted	83 (40.5)	4 (12.1)	
Item 13. Did the review authors account for RoB in individual studies when interpreting/discussing the results of the review? (n, %)			0.023
Yes	91 (44.4)	22 (66.7)	
No	114 (55.6)	11 (33.3)	
Item 14. Did the review authors provide a satisfactory explanation for, and discussion of, any heterogeneity observed in the results of the review? (n, %)			0.015
Yes	102 (49.8)	24 (72.7)	
No	103 (50.2)	9 (27.3)	
Item 15. If they performed quantitative synthesis did the review authors carry out an adequate investigation of publication bias (small study bias) and discuss its likely impact on the results of the review? (n, %)			0.841
Yes	64 (31.2)	11 (33.3)	
No/ No meta-analysis conducted	141 (68.8)	22 (66.7)	
Item 16. Did the review authors report any potential sources of conflict of interest, including any funding they received for conducting the review? (n, %)			0.388
Yes	179 (87.3)	31 (93.9)	
No	26 (12.7)	2 (6.1)	
Score: mean (SD)	14.5 (3.8)	18.0 (3.2)	0.000

PICO, specification of inclusion criteria including the population, intervention, comparison, outcome; RoB, risk of bias.

Supplementary Table 5. Details of PRISMA Assessment

PRISMA items	Nonregistered (n=205)	Registered (n=33)	*P*-value
Item 1. Title (n, %)			0.288
Yes	80 (39.0)	17 (51.5)	
Partial	116 (56.6)	16 (48.5)	
No/ Can't answer	9 (4.4)	0 (0.0)	
Item 2. Structured summary (n, %)			0.155
Yes	0 (0.0)	1 (3.0)	
Partial	203 (99.0)	32 (97.0)	
No/ Can't answer	2 (1.0)	0 (0.0)	
Item 3. Rationale (n, %)			—
Yes	205 (100.0)	33 (100.0)	
Partial	0 (0.0)	0 (0.0)	
No/ Can't answer	0 (0.0)	0 (0.0)	
Item 4. Objectives (n, %)			0.886
Yes	73 (35.6)	11 (33.3)	
Partial	130 (63.4)	22 (66.7)	
No/ Can't answer	2 (1.0)	0 (0.0)	
Item 5. Protocol and registration (n, %)			0.000
Yes	0 (0.0)	29 (87.9)	
Partial	24 (11.7)	4 (12.1)	
No/ Can't answer	181 (88.3)	0 (0.0)	
Item 6. Eligibility criteria (n, %)			0.806
Yes	50 (24.4)	6 (18.2)	
Partial	148 (72.2)	26 (78.8)	
No/ Can't answer	7 (3.4)	1 (3.0)	
Item 7. Information sources (n, %)			0.860
Yes	188 (91.7)	32 (97.0)	
Partial	7 (3.4)	0 (0.0)	
No/ Can't answer	10 (4.9)	1 (3.0)	
Item 8. Search (n, %)			0.861
Yes	183 (89.3)	2 (6.1)	
Partial	4 (2.0)	0 (0.0)	
No/ Can't answer	18 (8.8)	31 (93.9)	
Item 9. Study selection (n, %)			0.207
Yes	91 (44.4)	20 (60.6)	
Partial	95 (46.3)	12 (36.4)	
No/ Can't answer	19 (9.3)	1 (3.0)	
Item 10. Data collection process (n, %)			0.011
Yes	32 (15.6)	10 (30.3)	
Partial	132 (64.4)	22 (66.7)	
No/ Can't answer	41 (20.0)	1 (3.0)	
Item 11. Data items (n, %)			0.365

Yes	11 (5.4)	3 (9.1)	
Partial	143 (69.8)	25 (75.8)	
No/ Can't answer	51 (24.9)	5 (15.2)	
Item 12. Risk of bias in individual studies (n, %)			0.140
Yes	105 (51.2)	19 (57.6)	
Partial	59 (28.8)	12 (36.4)	
No/ Can't answer	41 (20.0)	2 (6.1)	
Item 13. Summary measures (n, %)			0.361
Yes	168 (82.0)	30 (90.9)	
Partial	7 (3.4)	1 (3.0)	
No/ Can't answer	30 (14.6)	2 (6.1)	
Item 14. Synthesis of results (n, %)			0.301
Yes	169 (82.4)	31 (93.9)	
Partial	2 (1.0)	0 (0.0)	
No/ Can't answer	34 (16.6)	2 (6.1)	
Item 15. Risk of bias across studies (n, %)			0.187
Yes	109 (53.2)	11 (33.3)	
Partial	0 (0.0)	0 (0.0)	
No/ Can't answer	96 (46.8)	22 (66.7)	
Item 16. Additional analyses (n, %)			0.006
Yes	19 (9.2)	3 (9.1)	
Partial	93 (45.4)	24 (72.7)	
No/ Can't answer	93 (45.4)	6 (18.2)	
Item 17. Study selection (n, %)			0.158
Yes	173 (84.4)	32 (97.0)	
Partial	17 (8.3)	0 (0.0)	
No/ Can't answer	15 (7.3)	1 (3.0)	
Item 18. Study characteristics (n, %)			0.093
Yes	91 (44.4)	11 (33.3)	
Partial	100 (48.8)	22 (66.7)	
No/ Can't answer	14 (6.8)	0 (0.0)	
Item 19. Risk of bias within studies (n, %)			0.001
Yes	108 (52.7)	28 (84.8)	
Partial	36 (17.6)	3 (9.1)	
No/ Can't answer	61 (29.8)	2 (6.1)	
Item 20. Results of individual studies (n, %)			0.291
Yes	143 (69.8)	27 (81.8)	
Partial	38 (18.5)	5 (15.2)	
No/ Can't answer	24 (11.7)	1 (3.0)	
Item 21. Synthesis of results (n, %)			0.395
Yes	156 (76.1)	26 (78.8)	
Partial	21 (10.2)	5 (15.2)	
No/ Can't answer	28 (13.7)	2 (6.1)	
Item 22. Risk of bias across studies (n, %)			0.749

Yes	102 (49.8)	18 (54.5)	
Partial	1 (0.4)	0 (0.0)	
No/ Can't answer	102 (49.8)	15 (45.5)	
Item 23. Additional analysis (n, %)			0.377
Yes	124 (60.4)	24 (72.7)	
Partial	12 (5.9)	2 (6.1)	
No/ Can't answer	69 (33.7)	7 (21.2)	
Item 24. Summary of evidence (n, %)			0.012
Yes	53 (25.9)	16 (48.5)	
Partial	152 (74.1)	17 (51.5)	
No/ Can't answer	0 (0.0)	0 (0.0)	
Item 25. Limitations (n, %)			0.094
Yes	38 (18.5)	4 (12.1)	
Partial	138 (67.3)	28 (84.8)	
No/ Can't answer	29 (14.1)	1 (3.0)	
Item 26. Conclusions (n, %)			0.840
Yes	142 (69.3)	22 (66.7)	
Partial	63 (30.7)	11 (33.3)	
No/ Can't answer	0 (0.0)	0 (0.0)	
Item 27. Funding (n, %)			0.111
Yes	78 (38.0)	5 (15.2)	
Partial	73 (35.6)	9 (27.3)	
No/ Can't answer	54 (26.3)	5 (15.2)	
Score: mean (SD)	17.6 (3.9)	20.4 (3.1)	0.000

Supplementary Table 6. Results of sensitivity analyses for variables associated with AMSTAR-2 scores and PRISMA scores

Variables	All items for AMSTAR-2				All items for PRISMA			
	Univariate		Multivariate		Univariate		Multivariate	
	Coef. (95% CI)	*P*	Coef. (95% CI)	*P*	Coef. (95% CI)	*P*	Coef. (95% CI)	*P*
Registered								
No	0		0		0		0	
Yes	2.31 (0.98, 3.65)	0.001	2.22 (0.96, 3.49)	0.001	1.99 (0.58, 3.40)	0.006	1.99 (0.67, 3.32)	0.003
Country or region								
USA/Canada	0		0		0		0	
Europe	0.08 (-1.14, 1.29)	0.900	0.02 (-1.16, 1.21)	0.968	-0.15 (-1.43, 1.13)	0.818	-0.29 (-1.53, 0.95)	0.649
China	2.41 (1.20, 3.63)	0.000	2.31 (1.11, 3.50)	0.000	2.74 (0.99, 4.50)	0.002	2.15 (0.90, 3.40)	0.001
Other Asian countries	3.31 (1.65, 4.97)	0.000	3.05 (1.43, 4.67)	0.000	2.43 (1.15, 3.72)	0.000	2.43 (0.74, 4.13)	0.005
Others	1.57 (-0.28, 3.42)	0.095	1.27 (-0.54, 3.07)	0.168	0.40 (-1.55, 2.36)	0.685	0.04 (-1.85, 1.93)	0.968
Journal impact factor	-0.03 (-0.10, 0.04)	0.431	---	---	0.06 (-0.01, 0.13)	0.106	---	---
No. of RCTs included	0.01 (-0.01, 0.02)	0.401	---	---	0.01 (-0.01, 0.02)	0.496	---	---
No. of patients included	0.00 (0.00, 0.00)	0.802	---	---	0.00 (0.00, 0.00)	0.148	---	---
Interventions								
Pharmacological	0		---	---	0		---	---
Operation	1.12 (-2.59, 4.83)	0.552	---	---	1.40 (-2.50, 5.29)	0.481	---	---
Psychological education	-0.34 (-4.60, 3.93)	0.877	---	---	-0.69 (-5.17, 3.79)	0.763	---	---
Disease management	-0.33 (-2.08, 1.42)	0.712	---	---	-0.08 (-1.92, 1.76)	0.933	---	---
Others	0.39 (-0.66, 1.45)	0.461	---	---	-0.30 (-1.41, 0.81)	0.594	---	---
Statistical result								
Negative	0		0		0		0	
Positive	1.10 (0.06, 2.13)	0.038	0.78 (-0.19, 1.75)	0.114	2.11 (1.05, 3.18)	0.000	1.81 (0.80, 2.83)	0.001

AMSTAR-2, Assessment of Multiple Systematic Review 2; PRISMA, Preferred Reporting Items for Systematic Reviews and Meta-analyses; Coef., coefficient; RCTs, randomized controlled trials.

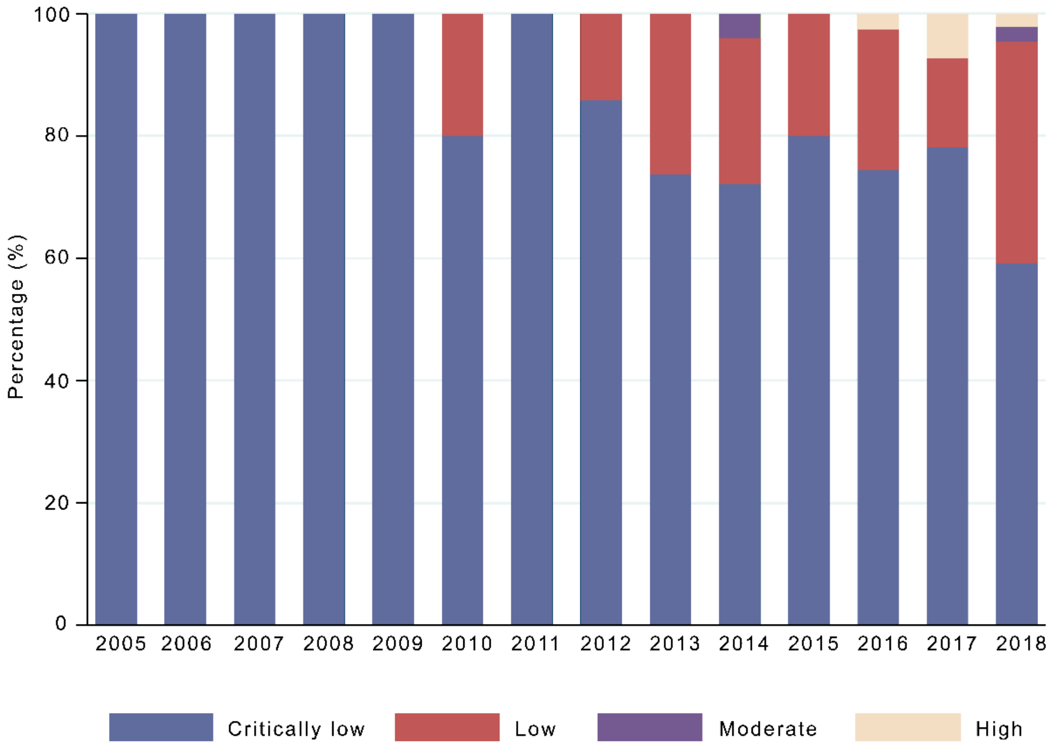

Supplementary Figure 1. Methodological quality of systematic reviews in type 2 diabetes mellitus between 2005-2018
